# Synthesis and Electronic Structure of a Tetraazanaphthalene Radical-Bridged Yttrium Complex

**DOI:** 10.1021/acsorginorgau.5c00086

**Published:** 2025-10-09

**Authors:** Saroshan Deshapriya, Selvan Demir

**Affiliations:** Department of Chemistry, 3078 Michigan State University, 578 South Shaw Lane, East Lansing, Michigan 48824, United States

**Keywords:** tetraazanaphthalene, radical, EPR spectroscopy, density functional theory, X-ray diffraction

## Abstract

Taming radical anions with highly electropositive metal ions poses a grand synthetic challenge owing to the high reactivity of such compounds originating from the unpaired electron. A successful synthetic metal radical match elicits a desire to thoroughly understand the electronic structure of a given metal radical pairing, which may inform about the potential physical properties pertaining to spintronics and magnetism relevant for future technologies. Here, the 1,4,5,8-tetraazanaphthalene (tan) ligand was utilized in the synthesis of (Cp*_2_Y)_2_(μ-tan), **1**, using the doubly reduced version K_2_tan and Cp*_2_Y­(BPh_4_) following a salt metathesis reaction. Chemical oxidation of **1** yielded [(Cp*_2_Y)_2_(μ-tan^•^)]­[BArF_20_], **2**, containing a tan^–•^ radical anion. **2** constitutes the first *d*-block coordination compound bearing a tan radical. **1** and **2** were studied through X-ray crystallography, electrochemistry, and spectroscopy. The radical nature of **2** was uncovered by cw-EPR spectroscopy and density functional theory (DFT) computations. All findings suggest major changes in the spin and charge distributions of this organic radical ligand when it is metalated. In fact, the results demonstrate that the tan^–•^ radical is more stable when coordinated to a transition metal than in its free nature, and thus, this insight is relevant for the development of future spintronic technologies.

## Graphical abstract

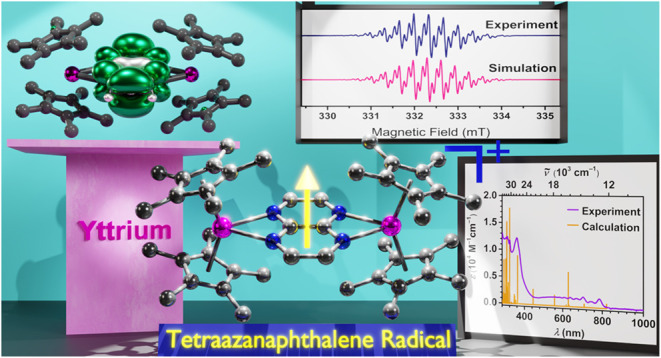

## Introduction

Redox-active ligands with accessible radical oxidation states occupy a crucial role in modern coordination chemistry owing to their prowess in enabling distinct electronic properties without necessitating changes to metal oxidation states.^1,2^ As ligated units, open-shell ligands can modulate frontier orbital energies, modify optical absorption features, and enhance magnetic communication between anisotropic paramagnetic metal centers, giving rise to applications in sensors,^3,4^ optoelectronic devices,^5,6^ and single-molecule magnets.^7–10^ Furthermore, the coordination of an organic radical to metal centers greatly enhances its stability, allowing the isolation of hitherto unprecedented radical oxidation states.^11,12^ Despite the broad utility of ligand-based redox chemistry, small, nitrogen-rich polycyclic frameworks that combine high electron affinity and a propensity to bridge metal centers remain relatively underexplored in coordination chemistry.

Nitrogen-substituted polycyclic aromatic compounds are especially attractive in this regard since the incorporation of nitrogen into the aromatic rings stabilizes low-lying π* orbitals, lowers reduction potentials, and thus, promotes ligand-centered reduction that is often made accessible under milder conditions.^13^ Within this class, 1,4,5,8-tetraazanaphthalene (tan) stands out as the smallest tetraazaacene, comprising two fused arene rings involving four N atoms.^14–16^ This scaffold facilitates examinations of azaacene topology governing reduction chemistry and electronic delocalization. Despite these appealing attributes and widespread theoretical interest in tetraazaacenes as compact electron acceptors,^17^ tan has not been extensively scrutinized as a bridging ligand in transition metal coordination chemistry. This is also reflected in the fact that structurally characterized *d*-block coordination compounds containing tan radicals have hitherto been unknown. Given that larger tetraaza ligands such as fluoflavine^11^ and bisbenzimidazole^18^ were demonstrated to function as tetradentate radical bridges in dinuclear metal complexes, the scrutiny of tan for this role is of particular interest. An appealing strategy is to bind the tan ligand to diamagnetic metal centers as a straightforward route to stabilize and interrogate ligand-centered redox events while simultaneously avoiding complicated metal-centered electron processes.^11,18,19^ To this end, the employment of yttrium­(III) ions to generate topologically similar complexes containing the tan ligand in dia- and paramagnetic states constitutes an ideal platform to study structure–property relationships and provides insight into the electronic structure of such compounds as a function of the tan oxidation state.

Herein, we describe the synthesis and characterization of the first yttrium complexes bearing tan in two chemically accessible ligand redox states. First, we isolated the diamagnetic dinuclear complex (Cp*_2_Y)_2_(μ-tan), **1**, (Cp* = pentamethycyclopentadienyl) comprising a doubly reduced tan dianion (tan^2–^). Second, a chemical oxidation of **1** led to the tan radical-bridged dinuclear yttrium complex, [(Cp*_2_Y)_2_(μ-tan^•^)]­[BArF_20_], **2**, ([BArF_20_ = tetrakis­(perfluorophenyl)­borate) bearing a tan radical monoanion (tan^–•^). **1** and **2** constitute the first *d*-block coordination compounds of tan. Both complexes were fully studied through single-crystal X-ray diffraction, infrared (IR), nuclear magnetic resonance (NMR), ultraviolet-visible (UV–vis) spectroscopy, and cyclic voltammetry. X-band electron paramagnetic resonance (EPR) spectroscopy unambiguously confirmed the radical nature of **2**, and density functional theory (DFT) calculations uncovered the electronic structures of both compounds. The use of yttrium­(III) as a diamagnetic metal center simplified spectroscopic interpretation tremendously and allowed the direct assignment of ligand-centered electronic changes upon reduction. Our findings establish tan as a compact, redox-active bridging ligand for the synthesis of coordination compounds involving *d*-block elements. Furthermore, the results provide a chemically simple, structurally authenticated platform for studying discrete ligand redox events and their impact on structural parameters and electronic structure in dinuclear assemblies.

## Experimental Section

### Experimental Materials and Methods

All manipulations were performed under an argon atmosphere with rigorous exclusion of oxygen and moisture using glovebox techniques, unless mentioned otherwise. House nitrogen was purified using an MBraun HP-500-MO-OX gas purifier. Tetrahydrofuran (THF) was refluxed over potassium for several days and subsequently dried further over a Na/K alloy. *n*-Hexane and 1,2-difluorobenzene (DFB) were dried over CaH_2_ for several days. The solvents (except for DFB) were tested for the presence of water and oxygen in the glovebox by the addition of one drop of potassium benzophenone radical solution to 2 mL of the solvent of interest. 2.2.2-cryptand was purchased from Sigma-Aldrich and recrystallized from hot hexane prior to use. [^n^Bu_4_N]­[PF_6_] was purchased from Sigma-Aldrich and recrystallized from THF prior to use. KCp*,^20^ (HNEt_3_)­(BPh_4_),^21^ Cp*_2_Y­(BPh_4_),^22^ Thianthrenium tetrakis­(perfluorophenyl)­borate (Thian^•^)­[BArF_20_],^23^ KC_8_,^24^ and 1,4,5,8-tetraazanapthalene (tan)^16^ were prepared according to literature procedures.

**Caution!** KC_8_ is a corrosive and extremely pyrophoric solid under ambient conditions. All manipulations were performed in an argon-filled MBRAUN glovebox with an atmosphere of <0.1 ppm of O_2_ and <0.1 ppm of H_2_O, and on a small practical scale following the procedures described below.

### Synthesis of (Cp*_2_Y)_2_(μ-tan), **1**

In an argon-filled glovebox, 11.0 mg (0.0833 mmol) of tan was weighed into a 7 mL scintillation vial charged with a magnetic stir bar, and subsequently, 3 mL of THF was added. To the stirring slurry was transferred 22.5 mg (0.1665 mmol) of KC_8_ in 2 mL of THF, which resulted in an immediate color change of the reaction mixture from pale yellow to dark blue. After stirring for 20 min at room temperature, the dark blue suspension was added at once to a stirring colorless solution of 112.9 mg (0.1665 mmol) of Cp*_2_Y­(BPh_4_) in 6 mL of THF in a 20 mL scintillation vial. An immediate color change of the reaction mixture from dark blue to dark red was observed. Stirring of the mixture was stopped after 4 h at room temperature, allowing the formed solids to settle. The red reaction mixture was filtered through a Celite plug into a fresh 20 mL vial to afford a clear red solution under removal of gray solids, presumably graphite and KBPh_4_. The solids were washed with ∼4 mL of THF, and the washings were filtered, and combined with the main red filtrate. The united solution was dried under vacuum to eliminate volatiles affording a bright red, powdery solid (crude yield: 51.5 mg, 73%). The obtained solid was extracted with 12 mL of toluene under stirring for 10 min producing a red toluene mixture which was filtered through Celite and dried under vacuum. The resulting bright red, powdery solid was extracted with 4 mL of toluene under stirring for 20 min at 80 °C yielding a dark red mixture which was filtered hot through Celite into a 7 mL vial. Subsequently, the clear red solution was allowed to cool down to room temperature and was further cooled to −35° overnight to produce bright red crystals of **1** suitable for single-crystal X-ray diffraction analysis. The crystals were separated from the mother liquor and washed with ∼2 mL of cold toluene prior to drying under vacuum for 2 h, yielding bright red crystalline material in 36.2 mg (51% crystalline yield). These crystals were further dried under vacuum for 4 h prior to elemental analysis. ^1^H NMR (500 MHz, ppm, C_6_D_6_, 25 °C): δ 5.00 (s, 4 H, C_6_ *H*_4_N_3_), 2.14 (s, 60 H, C_5_ *Me*_5_). ^13^C­{^1^H} NMR (126 MHz, ppm, C_6_D_6_, 25 °C) δ 123.3 (C_2_(*C* HN)_4_), 116.9 (*C*_5_Me_5_), 10.9 (C_5_ *Me*_5_). IR (FTIR, cm^–1^): 2855 (s), 2320 (vw), 2065 (vw), 1770 (w), 1627 (vw), 1541 (m), 1408 (vs), 1170 (vs), 1020 (s), 880 (w), 750 (vs). Anal. Calcd for C_46_H_64_N_4_Y_2_: C 64.94, H 7.58, N 6.58. Found: C 64.74, H 7.73, N 6.50.

### Synthesis of [(Cp*_2_Y)_2_(μ-tan^•^)]­[BArF_20_], **2**

In an argon-filled glovebox, 22.2 mg (0.0261 mmol) of **1** was weighed into a 20 mL scintillation vial charged with a magnetic stir bar. After the addition of 5 mL of DFB, and stirring for ∼5 min, all solids dissolved and produced a red solution. 28.0 mg (0.0313 mmol) of (Thian^•^)­[BArF_20_] was weighed into a 4 mL vial and dissolved into 2 mL of DFB to give a dark purple solution which was added at once to the stirring red solution of **1**, resulting in an immediate color change to black. The reaction mixture was stirred for 20 min and then dried under vacuum to afford a black solid residue. The solid was washed four times with each ∼4 mL of toluene portions, where the color of the pale orange wash solution presumably stemmed from unreacted **1**. Subsequently, the black solid was dried under vacuum for 1 h (crude yield: 32.7 mg, 82%), and then dissolved in 1.5 mL of DFB and filtered through a Celite plug into a 4 mL scintillation vial, prior to being carefully layered with 1.5 mL of *n*-Hexane. Black crystals of **2** suitable for single-crystal X-ray diffraction analysis were grown over 3 days at −35 °C from layering 1.5 mL of concentrated DFB solution with 1.5 mL of *n*-hexane in 73% crystalline yield. The crystals were separated from the mother liquor, washed with 1 mL of cold DFB, and subsequently dried under vacuum for 2 h. IR (FTIR, cm^–1^): 3657 (vw), 3584 (vw), 2912 (m), 2343 (vw), 1642 (s), 1459 (vs), 1268 (s), 1197 (s), 1084 (vs), 977 (vs), 755 (s), 658 (s). Anal. Calcd for C_70_H_64_N_4_Y_2_BF_20_: C 54.96, H 4.22, N 3.66. Found: C 54.68, H 4.13, N 3.49.

### Single-Crystal X-ray Diffraction (SCXRD)

Bright red, block-shaped crystals of **1** with dimensions of 0.239 × 0.145 × 0.085 mm^3^ and black, needle-shaped crystals of **2** with dimensions of 0.146 × 0.098 × 0.047 mm^3^ were mounted on a nylon loop using Paratone oil. Data was collected on a XtaLAB Synergy, Dualflex, and HyPix diffractometer equipped with an Oxford Cryosystems low-temperature device, operating at *T* = 100.00(10) and 100.00(11)­K, for **1** and **2**, respectively. Data for both crystals were measured by using ω scans implementing Mo and Cu Kα radiation for **1** and **2**, respectively. The total number of runs and images was based on the strategy calculation from the program CrysAlisPro (Rigaku, V1.171.41.90a, 2020),^25^ which was used to retrieve and refine the cell parameters as well as for data reduction. A numerical absorption correction based on Gaussian integration over a multifaceted crystal model empirical absorption correction using spherical harmonics was implemented in the SCALE3 ABSPACK scaling algorithm.^26^ The structures were solved in the *I*2/*a* space group for **1** and *P*1̅ space group for **2** using intrinsic phasing with the ShelXT structure solution program.^27^ The structure was refined by least-squares using version 2018/2 of XL^28^ incorporated in Olex2.^29^ All non-hydrogen atoms were refined anisotropically. Hydrogen atom positions were calculated geometrically and refined using the riding model. Crystals used for diffraction analysis showed no visible signs of decomposition under an optical microscope.

### UV–Vis Absorption Spectroscopy

The UV–vis spectra were recorded with an Agilent Cary 60 spectrometer at ambient temperature from 220 to 1000 nm. Samples were prepared in an argon-filled glovebox at 26 μM concentration of **1** and 27 μM concentration of **2** in THF and DFB, respectively, and filtered into 1 cm quartz cuvettes fitted with Schlenk adapters. The spectra were baseline corrected from blank samples of dry THF and DFB, respectively.

### FTIR Spectroscopy

Fourier transform infrared (FTIR) spectra were collected with an Agilent Cary 630 FTIR spectrometer on crushed crystalline solids of **1** and **2** under an inert nitrogen atmosphere.

### Elemental Analysis

Elemental analysis was carried out with a PerkinElmer 2400 Series II CHNS/O analyzer. The crystalline materials of **1** and **2** (∼1–3 mg) were weighed into tin sample holders and folded multiple times to ensure proper sealing under an argon atmosphere. The samples were then transferred to the instrument under exclusion of air in a sealed container.

### NMR Spectroscopy

NMR spectra were recorded on a Bruker Avance III HD 500 MHz NMR spectrometer in benzene-*d*_6_. NMR samples were prepared under an argon atmosphere. Benzene–*d*_6_ was purchased from Sigma-Aldrich and dried over molecular sieves prior to use.

### Cyclic Voltammetry

All cyclic voltammograms were taken under an argon-atmosphere. Measurements were performed by employing a Metrohm Autolab PGSTAT204 potentiostat with a glassy carbon working electrode, silver wire reference electrode, and platinum coil counter electrode. **1** and **2** were dissolved in 220 mM solutions of [*^n^* Bu_4_N]­[PF_6_] in DFB. The voltammograms were referenced internally to the ferrocene redox couple. Prior to each measurement, the glassy carbon working electrode was polished manually on an alumina slurry on wet sanding paper (1500 grit) by using a figure-8 motion. The electrode was then sonicated thoroughly with deionized water and 2-propanol (10 min each) to remove any residual abrasive particles.

### EPR Spectroscopy

EPR spectra were collected on a Bruker EMX–plus spectrometer operating at X-band (9.32 GHz) frequencies. The spectrometer is equipped with a Bruker ER4119HS probe and a modified Bruker liquid nitrogen variable-temperature accessory. A ∼3 mM solution of **2** was prepared in DFB and filled into a 3 mm OD quartz EPR tube. The cw-EPR spectrum was recorded under 24 dB microwave attenuation (0.7962 mW microwave power) and a 0.05 G modulation amplitude at room temperature. The spectrum taken was baseline corrected by using Xepr software prior to simulation.

### DFT Calculations

Density functional theory (DFT) computations were carried out for **1** and **2** using ORCA 5.0.4 software.^30,31^ Geometry optimization of both compounds was performed using uTPSSh functional^32,33^ with D3BJ dispersion correction^34,35^ at the def2-TZVP level.^36,37^ Vibrational frequencies were conducted on the optimized coordinates of both compounds by employing the same theory level. TD-DFT calculations were performed on the optimized structures of **1** and **2** with a CPCM THF solvent model and a manually defined DFB solvent model,^38^ respectively, using the uB3LYP functional^39,40^ for 150 excited states. All calculations employed the SARC/J auxiliary basis set.^41,42^ The generation of spin densities and molecular orbitals was accomplished using the orca_plot module, and the VMD program^43^ was employed for visualizations. Electronic transitions calculated through TD-DFT were shifted by 0.37 eV for **1** and 0.49 eV for **2** to coincide better with the experimental data.

## Results and Discussion

### Synthesis and Structural Characterization

Inspired by our isolation and first structural characterization of tan and tan^–•^ radical oxidation states,^16^ we set out to explore the complexation of tan^2–^ with the rare earth element yttrium. In particular, bridging two metallocene yttrium moieties of the type [Cp_2_^R^Y]^+^ (R = Me, H) through multidentate, redox-active, nitrogen-based ligands turned out to be lucrative when it comes to isolable organometallic products that inform about spin and charge density changes traversing from dia- to paramagnetic congeners.^11,18^ To this end, first K_2_tan was generated by the reduction of tan with two equivalents of KC_8_ and then used *in situ* in a salt metathesis reaction with Cp*_2_Y­(BPh_4_) (Cp* = pentamethylcyclopentadienyl) to form (Cp*_2_Y)_2_(μ-tan), **1**, accompanied by the byproduct KBPh_4_ that was removed through filtration. **1** was obtained as bright red, block-shaped crystals in 51% yield.

Complex **1** crystallizes in the monoclinic *I*2/*a* space group and comprises two yttrium­(III) ions, each capped by two Cp* ancillary ligands displaying η^5^ interactions. The tetradentate tan^2–^ ligand bridges the metal centers involving all four nitrogen atoms (Figure 1). The formula unit features a cocrystallized THF molecule that is disordered over two parts. The asymmetric unit consists of half a molecule **1**, i.e., one [Cp*_2_Y]^+^ moiety ligated to half of tan^2–^, and one part of the disordered THF (Figure S4). Thus, **1** contains an inversion center that is positioned on the central C–C bond of the coordinated tan^2–^.

**Figure 1:**
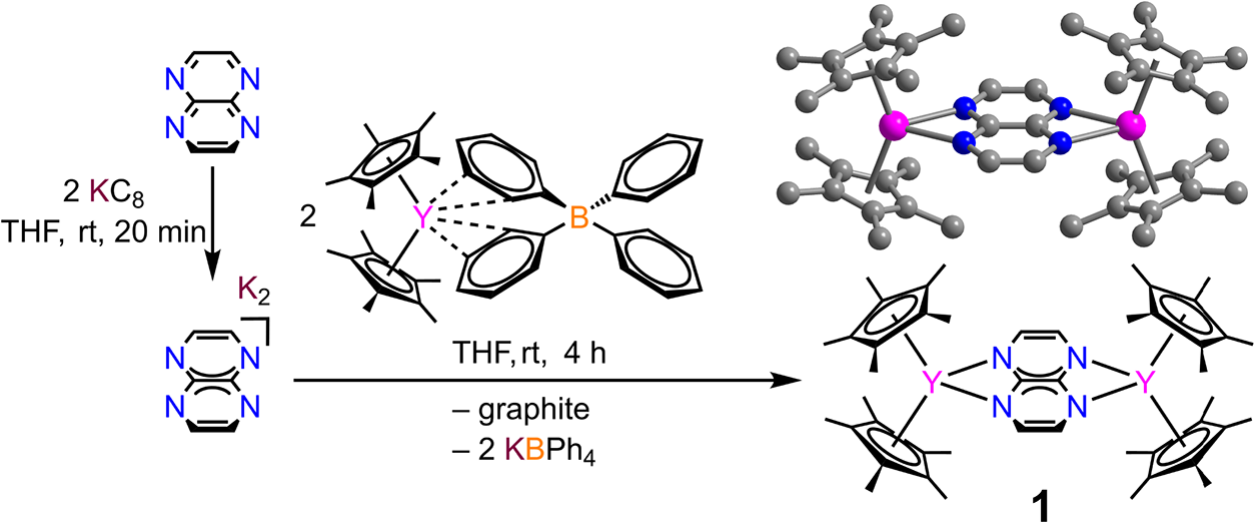
Synthesis of K_2_tan (left) and (Cp*_2_Y)_2_(μ-tan), **1**. Molecular structure of **1** in a crystal of (Cp*_2_Y)_2_(μ-tan)•C_4_H_8_O (top right). Pink, blue, and gray spheres represent Y, N, and C, respectively. H atoms and cocrystallized THF solvent molecule have been omitted for clarity.

The tan^2–^ entity bridging the two yttrium­(III) centers is completely planar where, in fact, all ligand atoms are in the same plane as the metal ions (Figure S9) and leads to a Y···Y distance of 7.004(4) Å. The Y–N interactions show an average distance of 2.377(2) Å, and the centroids of the Cp* ligands are situated at an average distance of 2.349(4) Å from the metal ions. These distances are slightly shorter compared to those of (Cp*_2_Y)_2_(μ-flv) (where flv = fluoflavine), representing a topologically similar complex relative to **1**, where flv constitutes an annulated version of tan by two additional peripheral phenyl rings.^11^ The latter compound features a Y···Y distance of 7.030(1) Å and an average Y–N distance of 2.387(2) Å. The 0.026 Å shorter intermetallic distance in **1** compared to that in (Cp*_2_Y)_2_(μ-flv) is ascribed to the decreased steric bulk arising from the lack of two phenyl rings in the tan ligand relative to the flv ligand. Similarly, the 0.01 Å decrease in the Y–N distances in **1** can be attributed to steric effects stemming from the compact tan ligand as opposed to a longer annulated version. The bond distances within the tan^2–^ unit are of interest upon coordination to metal ions when compared to the structures of unbound neutral tan^0^ and tan^–•^ radicals.^16^ The C–N bonds within the tan^2–^ unit of **1** exhibit an average distance of 1.371(3) Å, and the central C–C bond is 1.440(3) Å. By contrast, the neutral tan^0^ ligand displays shorter C–N and central C–C bond distances, where the average C–N distance is 1.337(1) Å and the central C–C bond distance is 1.415(1) Å. The respective distances for the tan^–•^ free radicals are 1.352(2) Å (C–N) and 1.448(3) (C–C) and 1.454(2) Å (C–C). Taken all into account, a general trend can be discerned for the C–N bond distances which rise with increasing charge on tan. Notably, the central C–C distance of tan^2–^ in **1** lies in between that of tan^0^ and tan^–•^ for which an explanation is provided by the bonding and antibonding nature of the orbitals involved, Figure 6.

The oxidation of **1** in DFB using the strong oxidant thianthrenium tetrakis­(perfluorophenyl)­borate, (Thian^•^)­[BArF_20_], results in the formation of [(Cp*_2_Y)_2_(μ-tan^•^)]­(BArF_20_), **2**, bearing a tan^–•^ radical anion (Scheme 1). Black crystals of **2** suitable for single-crystal X-ray diffraction analysis were isolated in 73% yield at −35 °C. **2** crystallizes in the *P*1̅ triclinic space group.

**Scheme 1:**
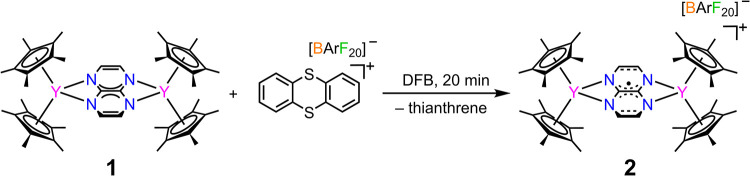
Synthesis of [(Cp*_2_Y)_2_(μ-tan^•^)]­[BArF_20_], **2**

**2** features two yttrium­(III) ions, each ligated η^5^ to two Cp* ligands, and which are bridged to one another through a tan^–•^ radical ligand (Figure 2). The asymmetric unit is composed of two half-fragments of **2**, i.e., two [Cp*_2_Y]^+^ units each ligated by half of a tan^–•^ fragment and a [BArF_20_]^−^ counteranion. (Figure S8). The two crystallographically independent molecular units exhibit subtle variations in bond metrics, which is attributed to their differing positions in the unit cell, where one is edge-centered and the other face-centered (Figure S6). For the discussion of these variants, the two units are labeled **2A** (edge-centered) and **2B** (face-centered) onward for clarity. A Cp* ring ligated to one of the yttrium­(III) centers of the **2A** molecule shows a π-interaction with an aryl ring of the [BArF_20_]^−^ counterion with a distance of 4.044(1) Å between the centroid of the Cp* moiety and the centroid of the aryl ring.

**Figure 2:**
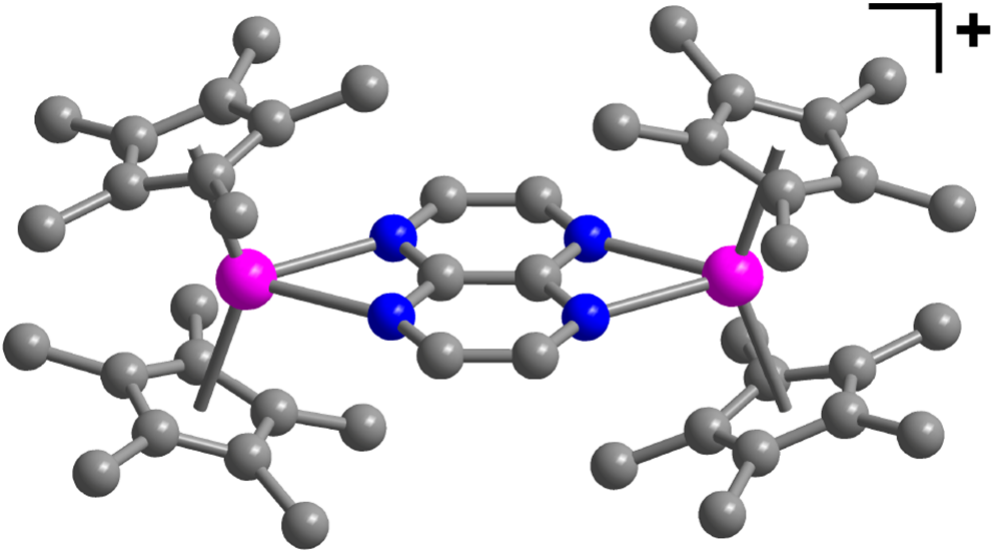
Molecular structure of [(Cp*_2_Y)_2_(μ-tan^•^)]^+^ in a crystal of **2**. Pink, blue, and gray spheres represent Y, N, and C, respectively. H atoms and the [BArF_20_]^−^ counteranion have been omitted for clarity.

Owing to the topological similarities, the bond metrics of **2A** and **2B** are compared to those of **1**. Complex **2** exhibits Y···Y distances of 7.108(8) and 7.138(8) Å for **2A** and **2B**, respectively. The average Y–N distances for **2A** and **2B** are 2.426(5) and 2.435(6) Å, respectively. Both of these bond distances are longer than the corresponding distances in **1**. This is attributed to the reduced negative charge of the tan^–•^ ligand in **2**, causing a weaker interaction between the yttrium­(III) centers and the bridging ligand. Conversely, the average yttrium–Cp* centroid distances are 2.324(4) and 2.319(5) Å in **2A** and **2B**, both being shorter than in **1**, which is ascribed to the reduced steric demand imposed by the weaker bound tan^–•^ unit owing to the smaller charge.

A bond metrics analysis of the tan^–•^ ligand in **2A** and **2B** uncovered the central C–C bond distances to be 1.413(8) and 1.414(8) Å, and the average C–N bond distances to be 1.356(7) and 1.359(8) Å. According to the trend unveiled for **1**, the C–N bond distance is shorter in tan^–•^ of **2**, further validating that these bonds elongate with increasing negative charge. The central C–C bond distance is virtually the same as that of the uncoordinated free tan^0^ compound.

Similar to the case of **1**, the tan^–•^ unit stays planar in both **2A** and **2B** (Figure S9). Furthermore, the bond distances of the tan^–•^ radical-bridged complex **2** can be compared to those of [(Cp*_2_Y)_2_(μ-flv^•^)]­[Al­(OC­{CF_3_}_3_)_4_], bearing the flv bridge in the −1 radical oxidation state.^11^ The flv^–•^ containing complex exhibits an intermetallic distance of 7.144(6) Å and an average Y–N distance of 2.437(2) Å. Similar to the metric changes observed on going from **1** to **2**, these distances increased traversing from a flv^2–^ to a flv^–•^ bridge.

### Electrochemical Analysis

The electrochemical behavior of **1** and **2** were probed via cyclic voltammetry. These experiments were conducted in DFB solutions with 3 mM analyte concentration and 220 mM [*^n^* Bu_4_N]­[PF_6_], supporting electrolyte concentrations. All cyclic voltammograms were collected under a 0.1 V/s scan rate, and internally referenced to the ferrocene redox couple.

The cyclic voltammogram of **1** was obtained via a negative to positive scan and exhibits two quasi-reversible features each with a half-step potential of −0.84 and −0.18 V (Figure 3). Both features are attributed to tan ligand-based redox events, corresponding to the tan^–•^/tan^2–^ and tan^0^/tan^–•^ redox couples, respectively. In addition, the two redox events can be separately produced through isolated potential scans, indicating that they can occur independently from each other (Figures S14 and S15).

**Figure 3:**
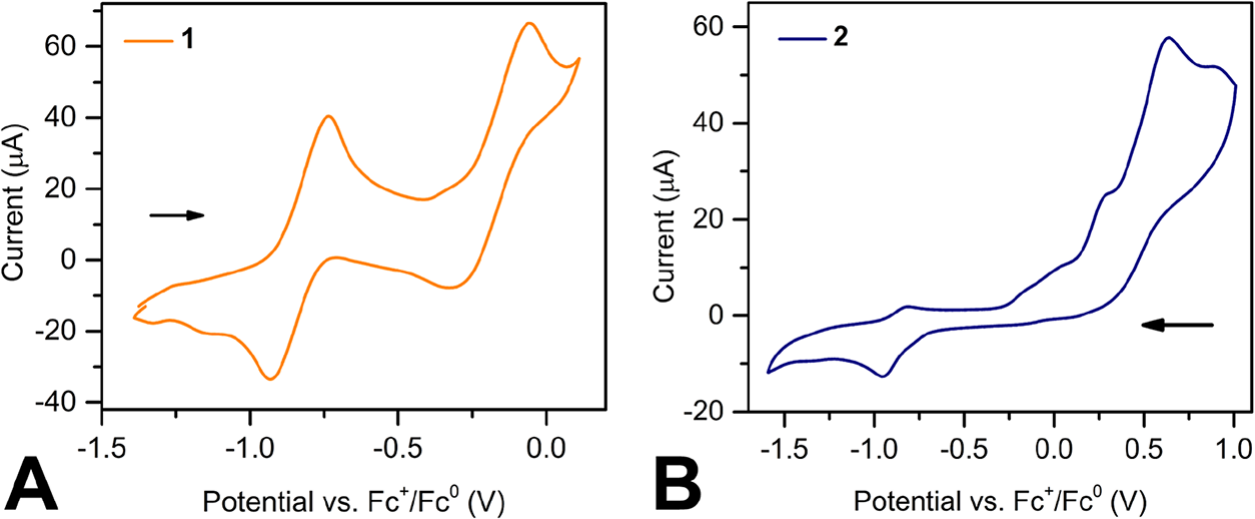
Cyclic voltammograms of (A) (Cp*_2_Y)_2_(μ-tan), **1**, and (B) [(Cp*_2_Y)_2_(μ-tan^•^)]­[BArF_20_], **2**, vs Fc^+^/Fc^0^ redox couple. Voltammograms were collected in DFB with a 220 mM concentration of [NBu_4_]­[PF_6_] supporting electrolyte and 3 mM analyte concentration. Arrows denote the voltage scanning direction. Cyclic voltammograms were plotted following polarographic convention.

In analogy to **1**, the cyclic voltammogram of **2** displays two quasi-reversible features when scanned from positive to negative potentials. These features bear half-step potentials at −0.89 and +0.16 V and correspond to the tan^–•^/ tan^2–^ and the tan^0^/ tan^–•^ redox couples, respectively. Relative to **1**, the features in **2** exhibit a positive shift in the potential. The more obvious positive shift observed for tan^0^/ tan^–•^ can be described from the energies of the frontier molecular orbitals (Figure S19). The singly occupied molecular orbital (SOMO) is significantly stabilized compared to the highest occupied molecular orbital (HOMO) of **1**, indicating the increased energy cost of removing an electron from the SOMO. In addition, during the potential scans, the color of the solution of **2** slowly and gradually changed from black to yellow, which is a sign of decomposition. This may be caused by the irreversible oxidative feature seen at around +0.64 V, which produces tan^0^ through the degradation of the metal complex.

The tan^–•^/ tan^2–^ redox event at −0.84 and −0.89 V against Fc^+^/Fc^0^ redox couple observed in the cyclic voltammograms of compounds **1** and **2** suggest that milder oxidants such as ferrocenium salts (*E*_1/2_ = 0 V against Fc^+^/Fc^0^ redox couple) may produce similar radical-containing complexes which will be studied in the future.

Additional electrochemical measurements were performed on **1** in THF (Figure S18). In comparison with the cyclic voltammogram collected in DFB, the tan^0^/ tan^–•^ redox event appears to be less reversible. This may be attributed to the coordinating ability of THF that may displace the oxidized tan ligand from the yttrium­(III) centers. Furthermore, the conducted variable scan rate measurements demonstrate that with a decreasing scan rate, both the peak-to-peak separation and the electrode current is reduced, suggestive of redox events being of quasi-reversible nature.

The redox events observed in cyclic voltammograms of **1** and **2** can be scrutinized against those observed for tan^–•^ radicals.^16^ The tan^–•^/ tan^2–^ and the tan^0^/ tan^–•^ redox events for the free radicals were found at −1.96 and −1.04 V potentials. Thus, upon coordination of the tan^2–^ ligand and the tan^–•^ radical species to the metal ions, the redox features shifted substantially toward positive potentials. This is ascribed to the stabilization of the SOMO of the tan^–•^ radical anion upon ligation to the yttrium­(III) centers. As the energies of the MOs are lowered, an oxidation becomes energetically less favorable, causing the redox events to move toward more positive potentials. This interpretation is in line with the observed positive shift of redox events in yttrium flv complexes compared to the flv^–•^ free radical.^11^

### Spectroscopic Analysis

FTIR spectroscopic measurements were conducted on both **1** and **2**. The collected spectra are innate to prominent vibrational modes (Figures S12 and S13). For both **1** and **2**, the features centered around ∼2900 and ∼1050 cm^–1^ correspond to twisting vibrations in the Cp* framework. For **2**, very strong features occur at 1084 and 977 cm^–1^, suggestive of the [BArF_20_]^−^ counteranion and thus, confirming the composition of **2** as the oxidation product that arose from **1**. These vibrational modes correspond to symmetric and antisymmetric C–F stretches that are absent for complex **1**.^44,45^

The diamagnetic nature of complex **1** enabled characterization through NMR spectroscopy, where the spectra were taken in benzene-*d*_6_. The ^1^H NMR spectrum exhibits two main peaks (Figure S10): first, a singlet is observed at 2.14 ppm that integrates to 60 protons and originates from the methyl protons of the Cp* ligands. Second, the four tan protons give rise to a singlet at 5.00 ppm. In addition, the cocrystallized THF is detected with peaks located at 1.41 and 3.59 ppm. The ^13^C NMR spectrum exhibits three main signals at 10.93, 116.89, and 123.26 ppm, representing the Cp* methyl carbons, Cp* ring carbons, and the peripheral carbons of the tan^2–^ unit, respectively. A comparison of the proton and carbon peak shifts in **1** and neutral tan^0^ reveals that upon complexation, the proton signal moved upfield by 4.25 ppm, and the peripheral carbon signal moved upfield by 26.75 ppm. This large shielding effect arises from the −2 negative charge of the tan unit.

UV–vis spectra were collected for **1** and **2** in a THF solution with a 26 μM analyte concentration and a DFB solution with a 27 μM analyte concentration, respectively (Figure 4). While THF allowed the collection of absorbance data from 1000 to 220 nm, the scan for **2** proceeded only down to 280 nm owing to the high absorption exhibited by DFB.

**Figure 4:**
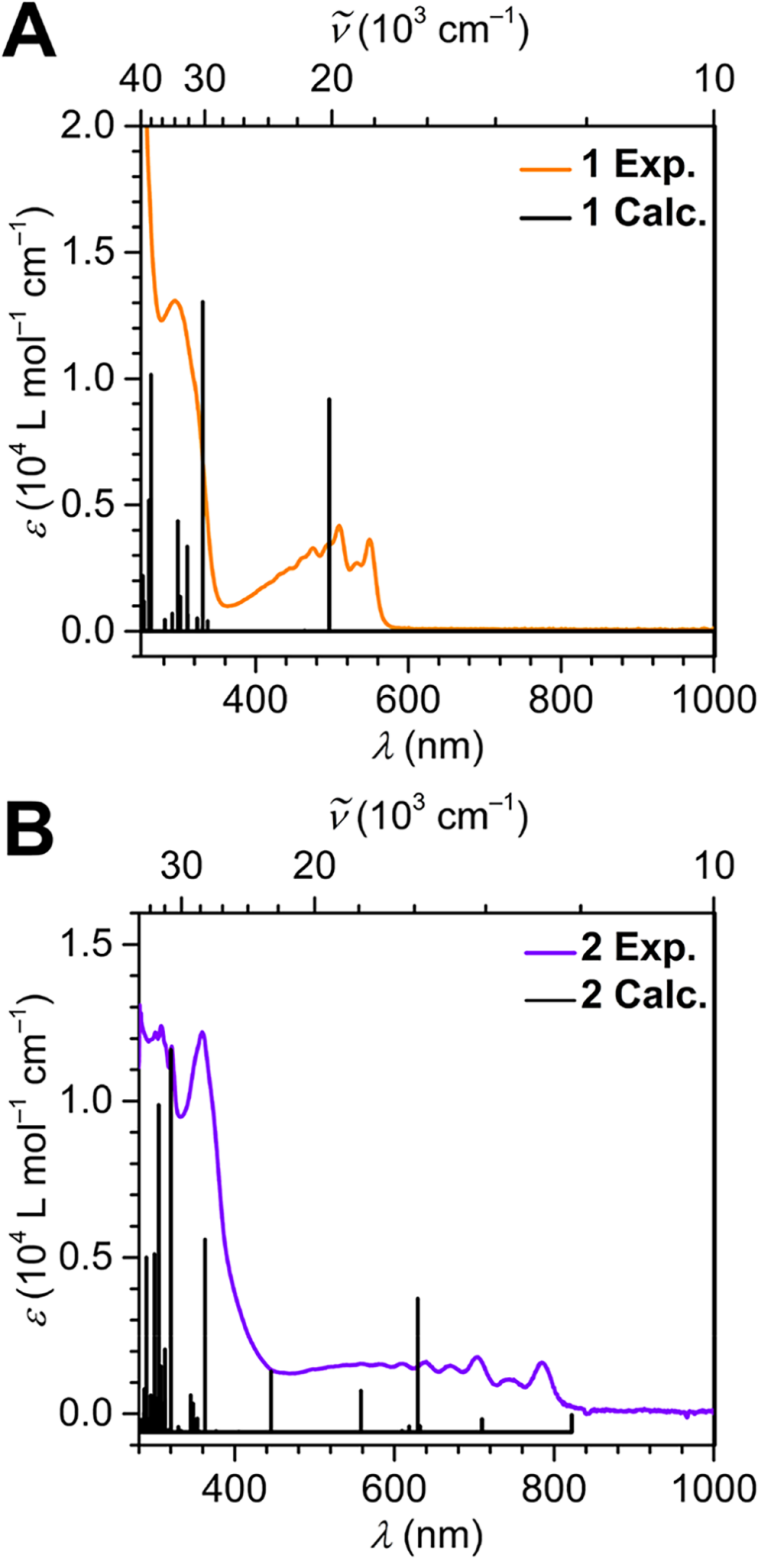
UV–vis spectra of (A) (Cp*_2_Y)_2_(μ-tan), **1**, at 26 μM concentration in THF and (B) [(Cp*_2_Y)_2_(μ-tan^•^)]­[BArF_20_], **2**, at 27 μM concentration in DFB. Orange and purple traces represent the experimental spectra for **1** and **2**, respectively. Black vertical lines are the predicted TD-DFT-predicted transitions.

The UV–vis absorption spectrum of **1** shows a low intensity feature with fine structure at 1.97 × 10^4^ cm^–1^ (508 nm). The most intense absorption feature in the visible region is broad and centered around 3.31 × 10^4^ cm^–1^ (302 nm), corresponding to absorption of violet-blue light. This is in agreement with the red color of the solution. The most prominent absorption is in the UV region with an intense peak at 4.77 × 10^4^ cm^–1^ (209 nm) with a shoulder peak at 4.11 × 10^4^ cm^–1^ (243 nm).

A similar low intensity feature with fine structure is visible in the UV–vis spectrum of **2**, centered at 1.42 × 10^4^ cm^–1^ (704 nm). The most intense absorption in the visible region occurs at 2.78 × 10^4^ cm^–1^ (359 nm). More fine structure is seen around absorption transpiring at 3.25 × 10^4^ cm^–1^ (307 nm). Overall, the features found in the spectra of compounds **1** and **2** are visually comparable. Yet, the energies corresponding to these transitions exhibit an apparent redshift in the case of **2**. This shift may arise from an additional stabilization predicted for the orbitals of **2** compared to **1**. Furthermore, the observed signals are similar to those seen for tan^–•^, albeit being less defined. The individual transitions involved in engendering these absorption features are discussed in the DFT section.

The radical nature of complex **2** was experimentally confirmed through cw-EPR spectroscopy. The EPR sample was prepared in DFB with an analyte concentration of ∼3 mM. Due to the high dielectric constant of DFB, a 3 mm OD quartz EPR tube was used to minimize microwave absorption by the solvent and the subsequent signal loss. The cw-EPR experiment was performed at X-band at room temperature with a 0.05 G modulation amplitude allowing the observation of a fine structure (Figure 5). The instrument was tuned at 9.32 GHz with a sweep width of 6 mT and a center field of 332.5 mT. The cw-EPR spectrum exhibits 11 main lines with additional splitting giving rise to fine structure. The spectrum was simulated with the Matlab toolbox EasySpin^46^ using the garlic module for solution samples. The simulation employed a *g* value of 2.0038, which is comparable to the free electron *g* value of 2.0023, indicative of an unpaired electron primarily distributed on the organic ligand with a small influence from the metal centers. Hence, for the simulation of the hyperfine couplings, ^14^N, ^1^H, and ^89^Y nuclei were considered.

**Figure 5:**
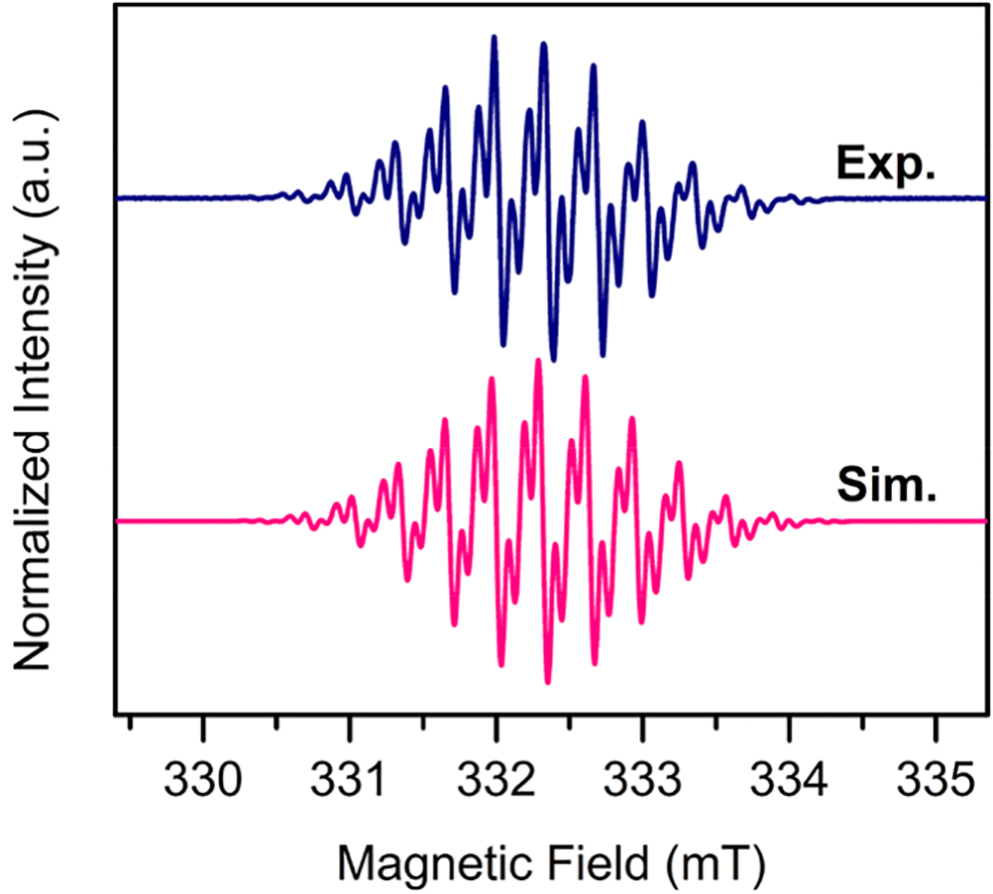
X-band cw-EPR spectrum of [(Cp*_2_Y)_2_(μ-tan^•^)]­[BArF_20_], **2**, collected in DFB at room temperature. Dark blue trace represents the experimental spectrum, and the pink trace constitutes the simulated spectrum. Parameters used for the simulation: Spin system of 4 ^14^N, 4 ^1^H, and 2 ^89^Y nuclei, *A*(^14^N) = 9.16 MHz, *A*_1_(^1^H) = 8.45 MHz, *A*_2_(^89^Y) = 2.76 MHz, *g* = 2.0038, line width (lw) = 0.065 mT.

The simulation takes into account 9.16 MHz hyperfine coupling constants arising from the four ^14^N nuclei and 8.45 MHz hyperfine coupling constants stemming from the four protons of the tan ligand. Mulliken spin population values generated through DFT calculations (Table S4) predict additional spin density on the two Y­(III) centers. Thus, two ^89^Y centers were additionally considered for simulating the spin system. To account for the molecular motion and the resultant averaging effects, a line broadening of 0.065 mT was utilized.

The spectrum of **2** shows similarities to the cw-EPR spectrum of the free tan^–•^ radical, exhibiting 11 main lines.^16^ However, owing to the presence of coordinating yttrium­(III) centers, the additional splitting engendered from the ^89^Y isotopes gives rise to more fine structure in the cw-EPR spectrum of **2**, contrasting it from that of the free tan^–•^ radical. This assignment further supports the DFT-predicted relative orbital energies, which originate from a delocalization of the unpaired electron beyond the tan ligand, causing the SOMO of **2** to be more stabilized compared to the HOMO of **1**.

Based on the parameters involved in simulating the cw-EPR spectrum, it can be deduced that the unpaired electron is strongly delocalized onto the N atoms, and therefore, the tan^–•^ radical anion interacts strongly with the coordinating yttrium­(III) centers. When extrapolated onto metal ions with highly anisotropic electronic distributions, this may engender strong magnetic exchange coupling between metal centers and the radical, which could be judiciously employed for advanced material design.

### DFT Computations

Density functional theory (DFT) calculations were employed to gain a deeper understanding of the electronic structures of **1** and **2**. For the geometry optimization of **2**, only the [(Cp*_2_Y)_2_(μ-tan^•^)]^+^ congener was considered, omitting the [BArF_20_]^−^ counteranion. All DFT calculations were carried out using ORCA 5.0.4 software.^31^ This process entails a geometry optimization of the crystal coordinates of **1** and **2** to attain energetic minimum structures as a first step. Accordingly, both structures were optimized using uTPSSh functional^32,33^ at the def2-TZVP level^36,37^ (Tables S4 and S5). The optimized structures were confirmed to be energetically minimum through the absence of imaginary frequencies in subsequent frequency calculations (Figures S12 and S13).

The geometry optimized structure of **1** exhibits an intermetallic distance of 6.975 Å and an average Y–N distance of 2.373 Å, while retaining the planarity of the tan ligand. The Cp* centroid to yttrium­(III) average distance is 2.345 Å confirming that the bond metrics of the optimized structure are comparable to those of the crystal structure.

Taking the ligated tan unit into consideration, the average C–N distance falls at 1.366 Å, while the central C–C bond length is 1.434 Å. These bond distances support an assignment of a −2 charge state on the tan ligand, where the majority of the negative charge is localized on the N atoms in accordance with their high electronegativity, Figure 6.

**Figure 6:**
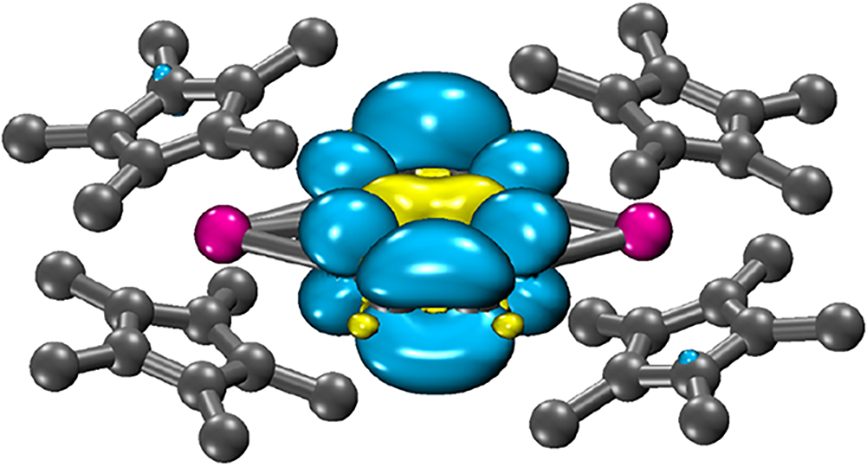
DFT-calculated spin density distribution of the [(Cp*_2_Y)_2_(μ-tan^•^)]^+^ moiety of [(Cp*_2_Y)_2_(μ-tan^•^)]­[BArF_20_], **2**. Pink, blue, and gray spheres represent the Y, N, and C atoms. H atoms have been omitted for the sake of clarity. Cyan and yellow surfaces represent different phases of spin density. Isovalues are set at 0.001 for depiction. Distribution of spin density surfaces precludes the visibility of N atoms. DFT theory level: uTPSSh, def2-TZVP and D3BJ approximation.

Similarly, the optimized structure of **2** displays a 7.139 Å Y···Y distance, an average Y–N distance of 2.445 Å, and an average Cp* centroid-yttrium­(III) distance of 2.321 Å. Congruent with the trend observed in the crystal structures of **1** and **2**, the intermetallic distance and the Y–N distance increased as the charge of the tan ligand was reduced, indicating weaker metal-tan coordination. Consequently, the monoanionic tan ligand imposes less steric bulk at the yttrium­(III) centers, enabling the Cp* rings to interact with the metal centers more strongly leading to shortened Cp* centroid-yttrium­(III) distances.

Analogously, the average C–N distance of the tan ligand in the optimized structure of **2** is 1.349 Å and the central C–C bond is 1.413 Å. The trajectory of increasing C–N distances as a function of a rising negative charge of the tan ligand holds validity when comparing the bond metrics of the optimized structures of **1** and **2**. Furthermore, the Mulliken charges evidence the charge state of the tan unit with the highest negative charges being accumulated on the ligating N atoms.

Frontier molecular orbitals were generated for **1** and **2** from their geometry optimized coordinates (Figure S19). The HOMO of **1** is mainly a tan ligand-based MO with contributions from C and N atomic orbitals. This shows π-bonding character, and no yttrium­(III) contribution can be discerned. The lowest unoccupied molecular orbital (LUMO) for **1** exhibits similar distribution over the tan ligand, with additional delocalization observed onto the metal centers and the Cp* framework. Contrasting to the HOMO, the MO distribution on tan adheres to an antibonding nature.

The HOMO of **2** is primarily Cp*-based with π-bonding character, while some delocalization onto the yttrium­(III) centers takes places. The SOMO is virtually identical in appearance to HOMO of **1** albeit some delocalization onto the Cp* rings occurs. The LUMO of **2** retains its topology akin to the LUMO of **1** and all three frontier orbitals of **2** are energetically stabilized relative to the HOMO of **1**. In comparison to the orbital energies of the free tan^–•^radical,^16^ the SOMO of **2** exhibits a substantially larger stabilization, indicative of the augmented stability of the radical upon coordination to two metal ions.

The most intense vibrational mode predicted by the frequency calculation for the optimized coordinates of **1** is a twisting motion involving all Cp* ligands at 2856 cm^–1^. The next largest mode is another twisting mode encompassing all of the Cp* ligands and the peripheral atoms of the tan unit. Another intense vibrational mode is found at 1308 cm^–1^ comprising a whole molecule asymmetric stretch. In the latter case, the stretch takes place along an axis that runs down the middle of the tan unit, bisecting the central C–C bond.

In contrast, for **2**, the strongest IR stretch is predicted to occur at 205 cm^–1^, beyond the accessible regime within the experimental spectrum. This strong IR stretch corresponds to a twisting mode of the Cp* framework. Akin to **1**, a Cp* framework twisting mode is also observed for **2** at 2859 cm^–1^, attesting to the similarity of the ancillary ligand scaffolds in both complexes. Furthermore, a whole molecule asymmetric stretch, akin to the one predicted for **1**, is monitored at 1347 cm^–1^, with a slight shift toward higher energies. Since this stretch is positioned around the central C–C bond of the ligated tan^–•^, it implies a strengthened bond upon oxidation, which is consistent with the shortened distance in the experimental crystal coordinates as well as in the geometry optimized structure.

The calculated time-dependent DFT (TD-DFT) transitions provide insight into the individual electronic excitations, giving rise to the observable features of the UV–vis spectra. The geometry optimized coordinates of **1** and **2** were employed in predicting the TD-DFT transitions by the use of the uB3LYP functional^39^ at the def2-TZVP level of theory. The calculated transitions were blueshifted by 0.37 and 0.49 eV for a better congruence with experimental spectra.

From the predicted individual transitions of **1**, the most intense absorbance is at 3.02 × 10^4^ cm^–1^ (331 nm) and corresponds to a HOMO–3, a molecular orbital with Cp* and yttrium­(III) bonding interactions, to LUMO transition. The next most intense feature is positioned in the UV region at 3.80 × 10^4^ cm^–1^ (263 nm), originating from a HOMO to LUMO+13 excitation. Here, the virtual orbital primarily consists of antibonding orbitals located at the Cp* framework, metal centers, and the tan ligand. The third highest absorption lies in the visible region at 2.01 × 10^4^ cm^–1^ (496 nm) and is ascribed to a HOMO to LUMO excitation.

Similarly, the strongest absorption feature of **2** constitutes a 3.12 × 10^4^ cm^–1^ (320 nm) excitation. Specifically, this feature corresponds to the excitation of electrons from HOMO–2 comprising Cp* and yttrium­(III) bonding orbitals to LUMO. The second most intense electronic absorption transition falls at 3.28 × 10^4^ cm^–1^ (305 nm) arising from a tan-centered bonding orbital, HOMO–9, to SOMO. The next most intense absorption is positioned at 2.75 × 10^4^ cm^–1^ (363 nm) reflecting a HOMO–2 to LUMO excitation. All excitations originate mainly from ligand-based orbitals, which is in agreement with an unpaired electron that is primarily delocalized on the bridging tan ligand. Detailed information about other TD-DFT transitions and the MOs involved in them are listed in Tables S2 and S3 of the Supporting Information.

## Conclusions

The first synthesis, isolation, and characterization of a *d*-block metal complex containing a 1,4,5,8-tetraazanaphthalene (tan) radical is reported. To this end, doubly reduced tan was employed *in situ* in a salt metathesis reaction with a suitable yttrium­(III) source to give (Cp*_2_Y)_2_(μ-tan), **1**, with a tan^2–^ bridge. **1** was oxidized to generate [(Cp*_2_Y)_2_(μ-tan^•^)]­[BArF_20_], **2**, bearing a tan^–•^ radical anion. The solid-state structures of **1** and **2** were characterized by single-crystal X-ray diffraction and revealed bond metrics that unambiguously confirmed the charge states of the bridging tan ligands.

NMR and FTIR spectroscopic characterizations affirmed the nature of each compound, whereas UV–vis analysis offered insight into the electronic structures of both complexes. X-band cw-EPR spectroscopy gave evidence of the presence of a tan^–•^ radical in **2** and provided insight into the distribution of the unpaired electron. Ultimately, EPR analysis revealed stabilization of the tan^–•^ radical through its coordination to the yttrium­(III) ions. DFT calculations supported the crystallographic and spectroscopic findings and provided in-depth information on the electronic properties of each complex. The spin density distribution proves the cw-EPR results and the TD-DFT calculations unravel the individual excitations contributing to the UV–vis absorption features.

In comparison to the free tan^–•^ radical, complex **2** is innate to an extended delocalization of the unpaired electron, resulting in an increased stabilization of the orbital energies. This, in turn, validates the proposition that the coordination of organic radicals to metal ions enhances their stability and, consequently, their applicability in spintronic technologies, paving the way for new frontiers in spin-based materials.

## Supplementary Material



## Data Availability

The data underlying this study are available in the published article and its Supporting Information.

## Notes

Crystallographic refinement parameters, NMR and IR spectra, cyclic voltammetry data, computational data, and coordinates of optimized structures (PDF)
