# Supplementary material for: Synthesis and Electronic Structure of a Tetraazanaphthalene Radical-Bridged Yttrium Complex
Source: ACS Org Inorg Au. 2025 Oct 9;5(6):557–66. doi: 10.1021/acsorginorgau.5c00086 (PMC12679307; doi:10.1021/acsorginorgau.5c00086)
Supplement: Supplementary file 1 [file gg5c00086_si_001.pdf]

Supporting Information

for

Synthesis and Electronic Structure of a Tetraazanaphthalene Radical-Bridged Yttrium Complex

Saroshan Deshapriya and Selvan Demir*

Department of Chemistry, Michigan State University, 578 South Shaw Lane, East Lansing, Michigan 48824, USA

*Correspondence to: sdemir@chemistry.msu.edu (S.D.)

Table of Contents

1 X-ray Crystallography **S4**

Table S1. Crystallographic data and structural refinements of **1** and **2**. **S4**

Figure S1. Thermal ellipsoid plot of $(Cp^*{}_2Y)_2$(μ-tan), **1**. **S5**

Figure S2. Unit cell of $(Cp^*{}_2Y)_2$(μ-tan), **1**. **S6**

Figure S3. Space-filling model of $(Cp^*{}_2Y)_2$(μ-tan), **1**. **S7**

Figure S4. Asymmetric unit of $(Cp^*{}_2Y)_2$(μ-tan), **1**. **S8**

Figure S5. Thermal ellipsoid plot of $[(Cp^*{}_2Y)_2(\mu\text{-tan}^{\bullet})][BArF_{20}]$, **2**. **S9**

Figure S6. Unit cell of $[(Cp^*{}_2Y)_2(\mu\text{-tan}^{\bullet})][BArF_{20}]$, **2**. **S10**

Figure S7. Space-filling model of $[(Cp^*{}_2Y)_2(\mu\text{-tan}^{\bullet})][BArF_{20}]$, **2**. **S11**

Figure S8. Asymmetric unit cell of $[(Cp^*{}_2Y)_2(\mu\text{-tan}^{\bullet})][BArF_{20}]$, **2**. **S12**

Figure S9. Side views of $(Cp^*{}_2Y)_2$(μ-tan), **1** and $[(Cp^*{}_2Y)_2(\mu\text{-tan}^{\bullet})][BArF_{20}]$, **2A** and **2B**. **S13**

2 NMR Spectroscopy **S14**

Figure S10. ^{1}H NMR spectrum of $(Cp^*{}_2Y)_2$(μ-tan), **1**. **S14**

Figure S11. $^{13}C\{^1H\}$ NMR spectrum of $(Cp^*{}_2Y)_2$(μ-tan), **1**. **S15**

3 IR Spectroscopy **S16**

Figure S12. FTIR spectrum of $(Cp^*{}_2Y)_2$(μ-tan), **1**. **S16**

Figure S13. FTIR spectrum of $[(Cp^*{}_2Y)_2(\mu\text{-tan}^{\bullet})][BArF_{20}]$, **2**. **S17**

4 Cyclic Voltammetry **S18**

Figure S14. Redox event observed at –0.84 V in the cyclic voltammogram of $(Cp^*{}_2Y)_2$(μ-tan), **1**, vs. Fc. **S18**

Figure S15. Redox event observed at –0.18 V in the cyclic voltammogram of $(Cp^*{}_2Y)_2$(μ-tan), **1**, vs. Fc. **S19**

Figure S16. Redox event observed at –0.89 V in the cyclic voltammogram of $[(Cp^*{}_2Y)_2(\mu\text{-tan}^{\bullet})][BArF_{20}]$, **2** vs. Fc. **S20**

Figure S17. Redox event observed at +0.16 V in the cyclic voltammogram of $[(Cp^*{}_2Y)_2(\mu\text{-tan}^{\bullet})][BArF_{20}]$, **2** vs. Fc. **S21**

Figure S18. Cyclic voltammograms of $(Cp^*{}_2Y)_2$(μ-tan), **1**, vs. Fc. in THF, using variable scan rates. **S22**

5 DFT Calculations **S23**

Figure S19. The DFT–calculated frontier molecular orbitals of $(Cp^*{}_2Y)_2$(μ-tan), **1**, and $[(Cp^*{}_2Y)_2(\mu\text{-tan}^{\bullet})][BArF_{20}]$, **2**. **S23**

Table S2. TD-DFT–calculated transition states for $(Cp^*{}_2Y)_2$(μ-tan), **1**. **S24**

Table S3. TD-DFT–calculated transition states for $[(Cp^*{}_2Y)_2(\mu\text{-tan}^{\bullet})][BArF_{20}]$, **2**. **S26**

Table S4. DFT–calculated Mulliken spin populations of $[(Cp^*{}_2Y)_2(\mu\text{-tan}^{\bullet})][BArF_{20}]$, **2**. **S28**

Table S5. Cartesian coordinates of the geometry optimized structure of $(Cp^*{}_2Y)_2$(μ-tan), **1**. **S29**
Table S6. Cartesian coordinates of the geometry optimized structure of $[(Cp^*{}_2Y)_2(\mu\text{-tan}^\bullet)][BArF_{20}]$, **2**. **S32**

1 X-ray Crystallography

Table S1. Crystallographic data and structural refinements of $(Cp^*{}_2Y)_2(\mu\text{-tan})$, **1**, and $[(Cp^*{}_2Y)_2(\mu\text{-tan}^{\bullet})][BArF_{20}]$. **1** crystallized with one THF solvent molecule in the lattice as $(Cp^*{}_2Y)_2(\mu\text{-tan})\bullet C_4H_8O$.

	**1**	**2**
Empirical formula	$C_{50}H_{72}N_4OY_2$	$C_{70}H_{64}BF_{20}N_4Y_2$
CCDC	2479235	2479236
Formula weight	922.93	1529.88
Temperature/K	100.00(10)	100.00(11)
Crystal system	monoclinic	triclinic
Space group	*I*2/*a*	*P*-1
a/Å	14.4645(3)	10.5236(2)
b/Å	16.4766(4)	12.6330(2)
c/Å	19.5002(4)	25.5314(5)
α/°	90	77.5143(16)
β/°	95.915(2)	79.0310(19)
γ/°	90	83.6682(16)
Volume/Å^3	4622.67(19)	3244.99(12)
Z	4	2
ρ_{calc}g/cm^3	1.326	1.566
μ/mm^{-1}	2.538	3.305
F(000)	1944.0	1550.0
Crystal size/mm^3	0.239 × 0.145 × 0.085	0.146 × 0.098 × 0.047
Radiation	Mo Kα (λ = 0.71073)	Cu Kα (λ = 1.54184)
2Θ range for data collection/°	4.944 to 57.726	7.366 to 136.48
Index ranges	-18 ≤ h ≤ 18, -21 ≤ k ≤ 21, -25 ≤ l ≤ 24	-12 ≤ h ≤ 12, -13 ≤ k ≤ 15, -30 ≤ l ≤ 30
Reflections collected	14692	42163
Independent reflections	5286 [R_{int} = 0.0348, R_{sigma} = 0.0372]	11836 [R_{int} = 0.0680, R_{sigma} = 0.0604]
Data/restraints/parameters	5286/75/290	11836/0/989
Goodness-of-fit on F^2	1.064	1.137
Final R indexes [I>=2σ (I)]	R_1 = 0.0432, wR_2 = 0.1129	R_1 = 0.0653, wR_2 = 0.1613
Final R indexes [all data]	R_1 = 0.0528, wR_2 = 0.1177	R_1 = 0.0774, wR_2 = 0.1670
Largest diff. peak/hole / e Å^{-3}	1.17/-0.51	2.26/-0.84

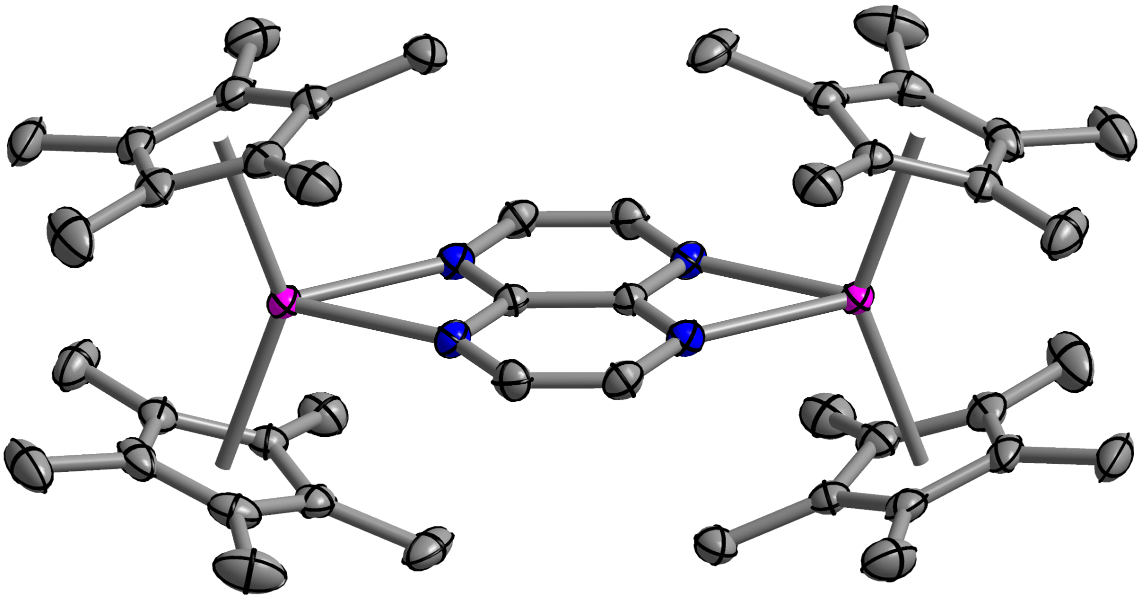

Figure S1. Structure of $(Cp^*{}_2Y)_2(\mu\text{-tan})$, **1**, in a crystal of $(Cp^*{}_2Y)_2(\mu\text{-tan})\bullet C_4H_8O$ with thermal ellipsoids drawn at the 50% probability level. Pink, blue, and gray spheres represent Y, N, and C atoms, respectively. H atoms and co-crystallized THF have been omitted for clarity.

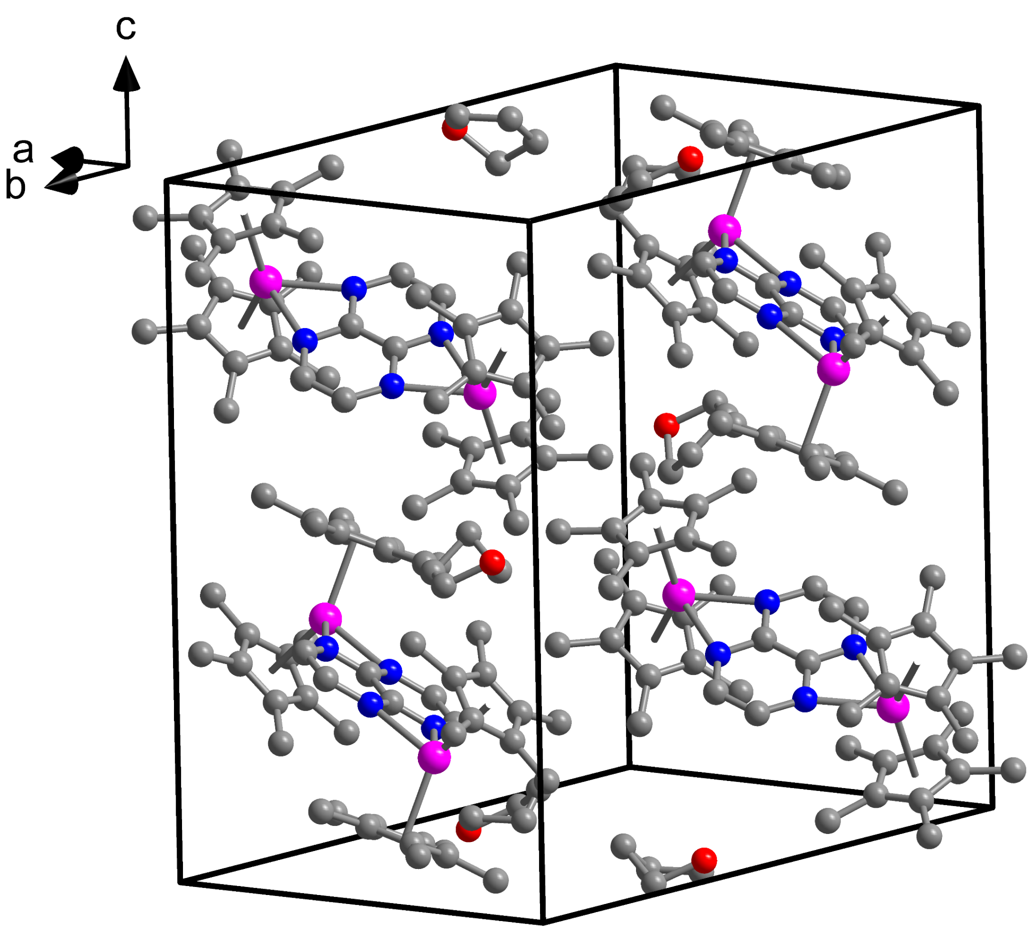

Figure S2. Unit cell of $(Cp^*_2Y)_2$(μ-tan), **1**, in a crystal of $(Cp^*_2Y)_2$(μ-tan)•C_4H_8O. Pink, red, blue, and gray spheres represent Y, O, N, and C atoms, respectively. H atoms have been omitted for clarity.

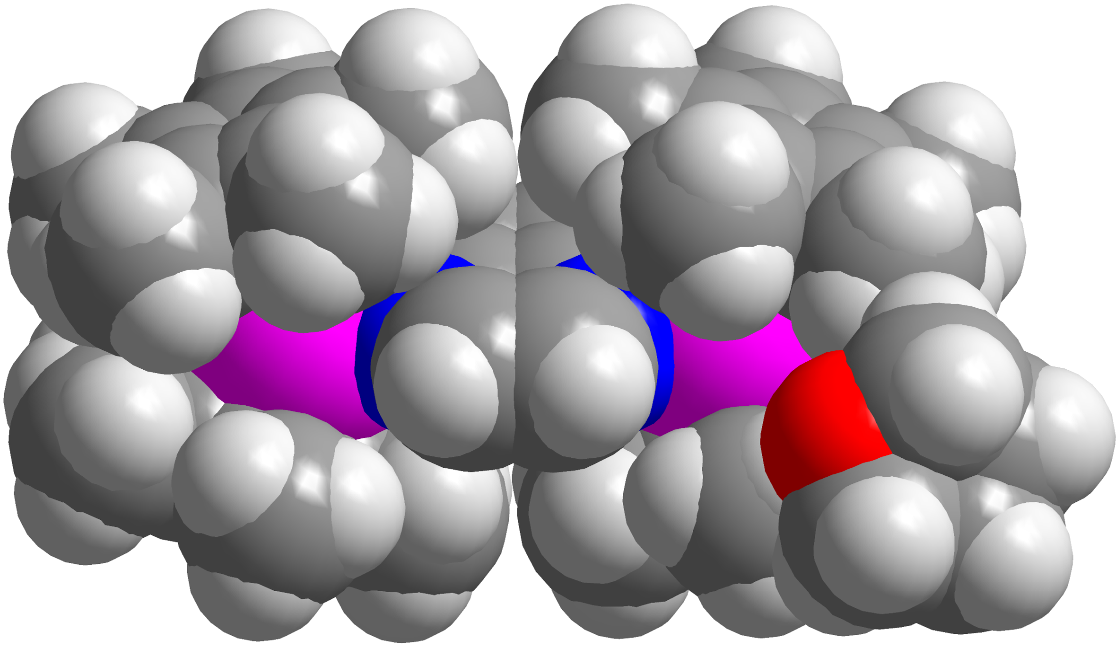

Figure S3. Space-filling model of $(Cp^*{}_2Y)_2$(μ-tan), **1**, in a crystal of $(Cp^*{}_2Y)_2$(μ-tan)•C_4H_8O. Pink, red, blue, gray, and white-gray spheres represent Y, O, N, C, and H atoms, respectively.

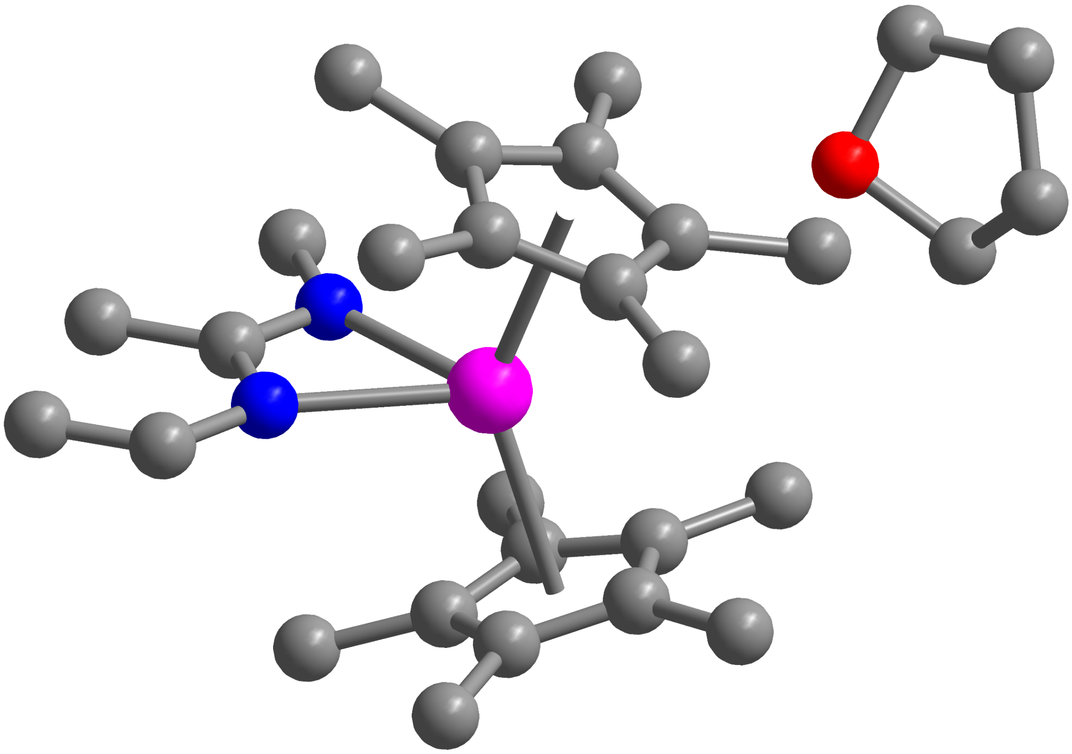

Figure S4. Asymmetric unit structure of the crystal of $(Cp^*_2Y)_2$(μ-tan)•C_4H_8O. Pink, red, blue, and gray represent Y, O, N, and C atoms, respectively. H atoms have been omitted for clarity.

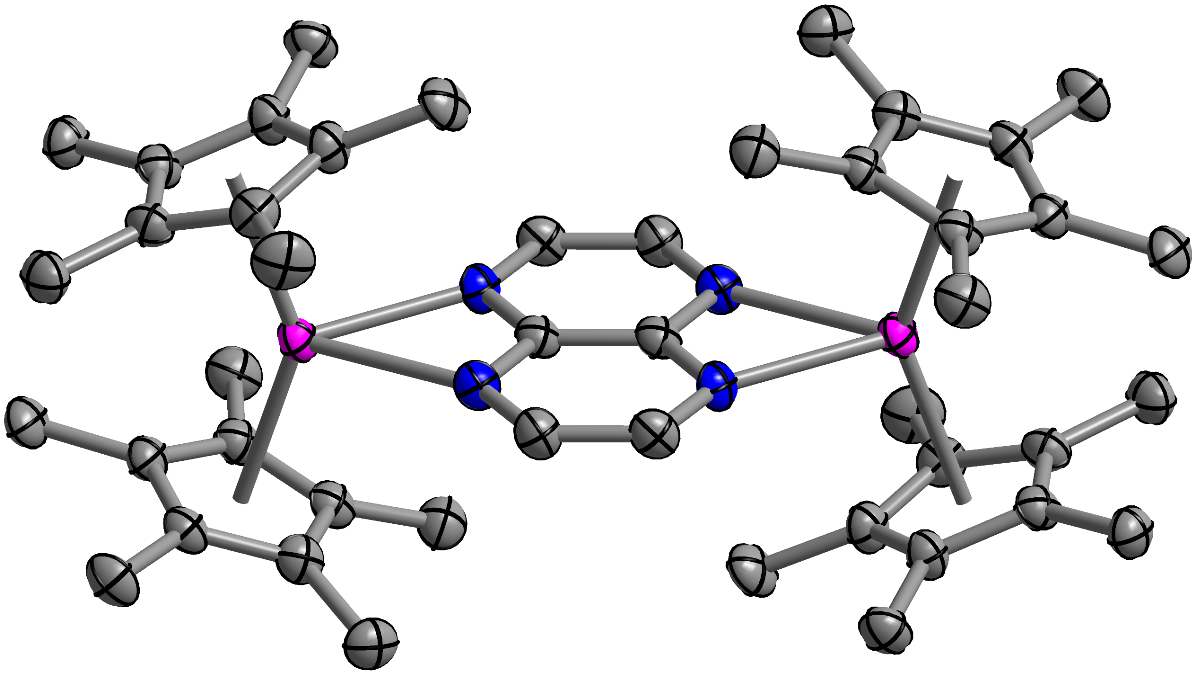

Figure S5. Structure of $[(Cp^*{}_2Y)_2(\mu\text{-tan}^{\bullet})]^+$ in a crystal of $[(Cp^*{}_2Y)_2(\mu\text{-tan}^{\bullet})][BArF_{20}]$, **2**, with thermal ellipsoids drawn at the 50% probability level. Pink, blue, and gray spheres represent Y, N, and C atoms, respectively. H atoms and $[BArF_{20}]^-$ counteranion have been omitted for clarity.

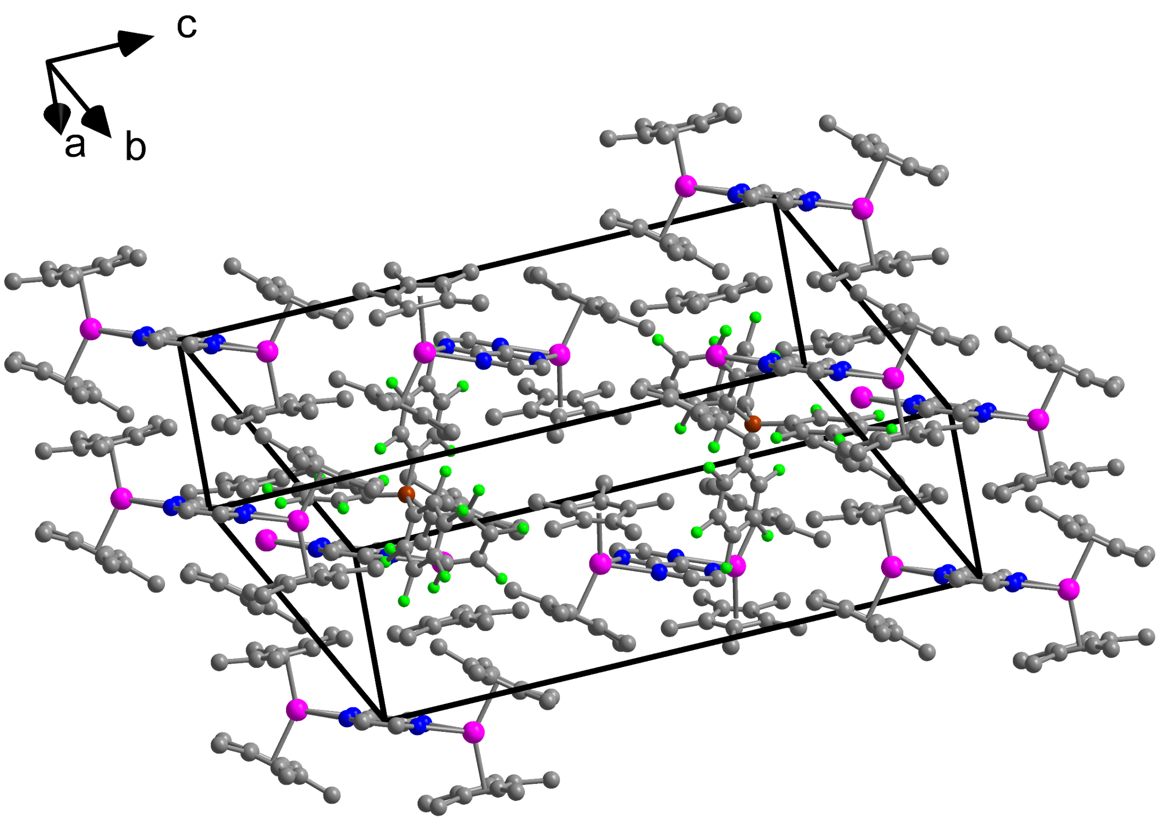

Figure S6. Unit cell of [(Cp*$_2$Y)$_2$(μ-tan$^•$)][BArF$_{20}$], **2**. Pink, green, blue, gray, and brown spheres represent Y, F, N, C, and B atoms, respectively. H atoms have been omitted for clarity.

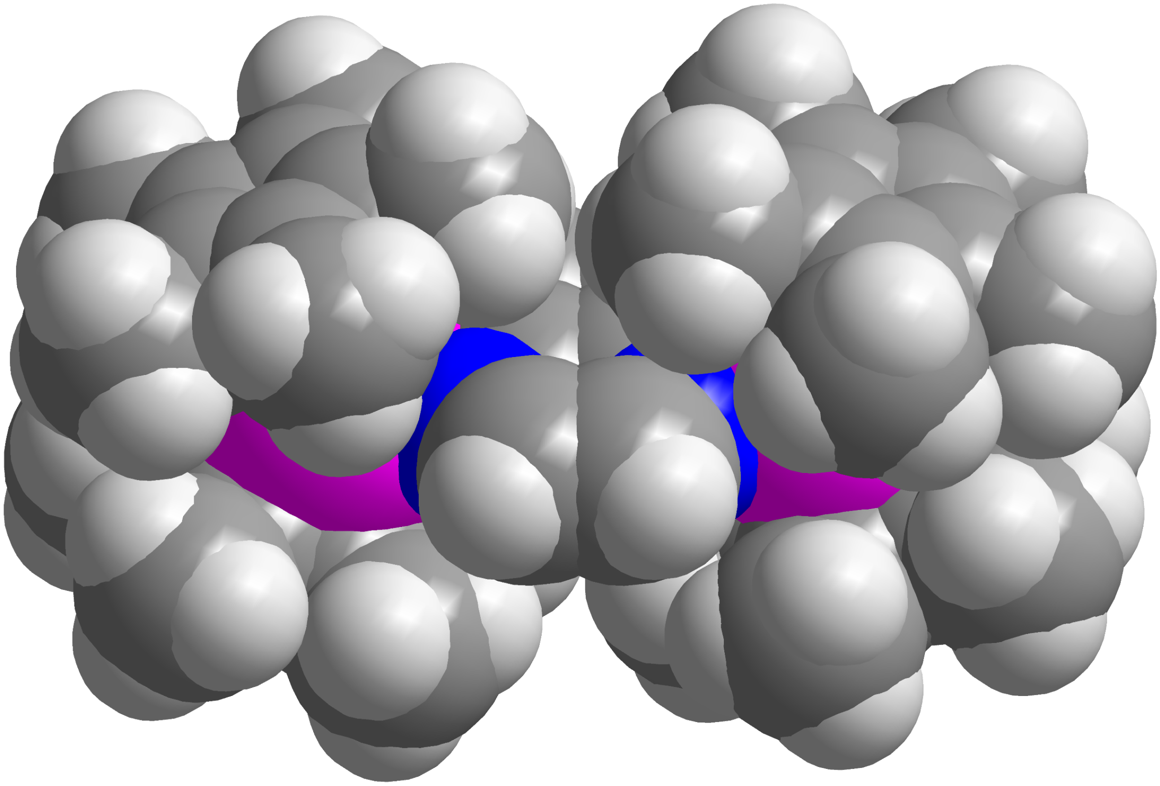

Figure S7. Space-filling model of $[(Cp^*_2Y)_2(\mu\text{-tan}^{\bullet})]^+$ in a crystal of $[(Cp^*_2Y)_2(\mu\text{-tan}^{\bullet})][BArF_{20}]$, **2**. Pink, blue, gray, and white-gray spheres represent Y, N, C, and H atoms, respectively. $[BArF_{20}]^-$ counteranion has been omitted for clarity.

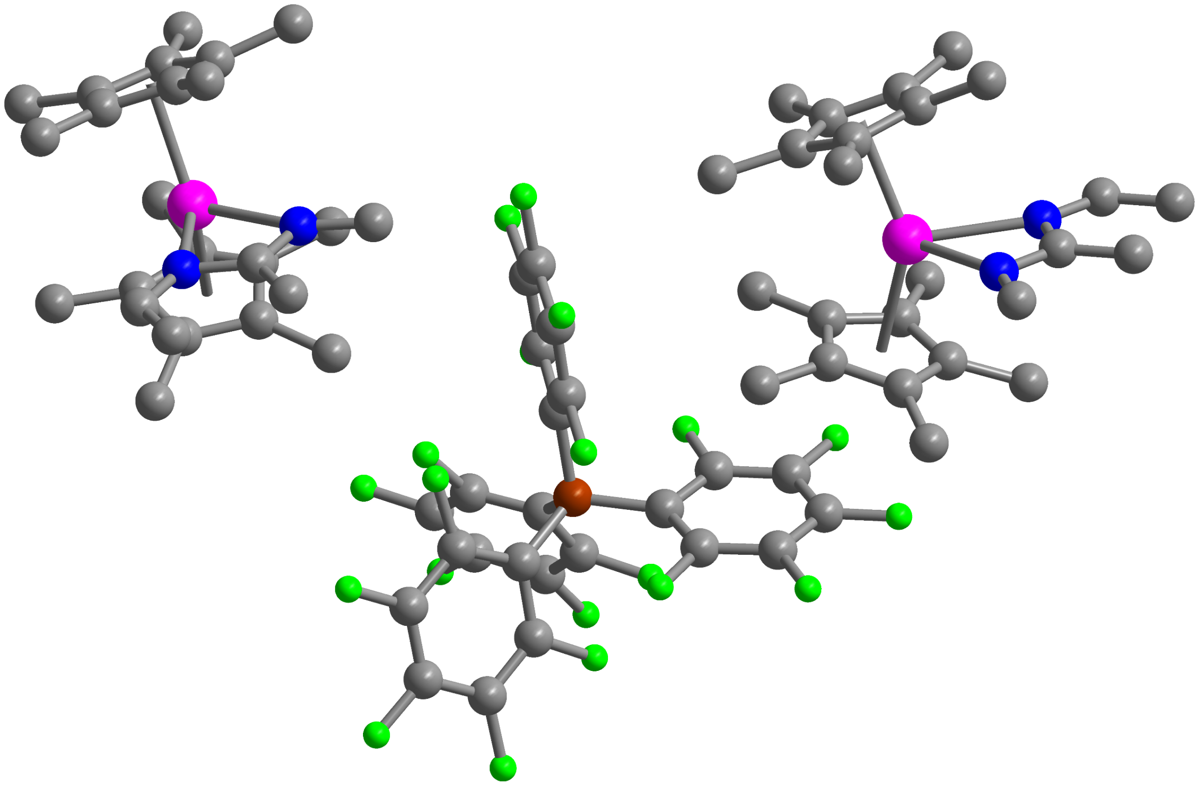

Figure S8. Asymmetric unit structure of the crystal of [(Cp*$_2$Y)$_2$(μ-tan$^{•}$)][BArF$_{20}$]. Pink, green, blue, gray, and brown spheres represent Y, F, N, C, and B atoms, respectively. H atoms have been omitted for clarity.

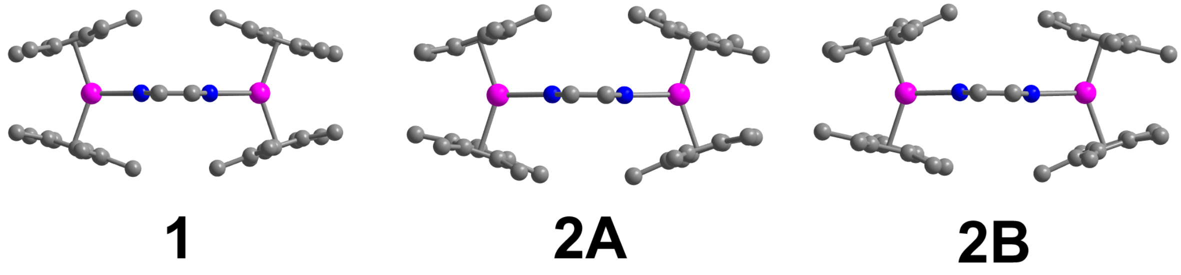

Figure S9. Side views of $(Cp^*_2Y)_2(\mu\text{-tan})$, **1**, and $[(Cp^*_2Y)_2(\mu\text{-tan}^{\bullet})][BArF_{20}]$, **2A** (edge-centered unit), **2B** (face-centered unit) showing the planarity of ligated tan in the complexes. Pink, blue, and gray spheres represent Y, N, and C atoms, respectively. H atoms, counterions, and co-crystallized solvent molecules have been omitted for clarity.

2 NMR Spectroscopy

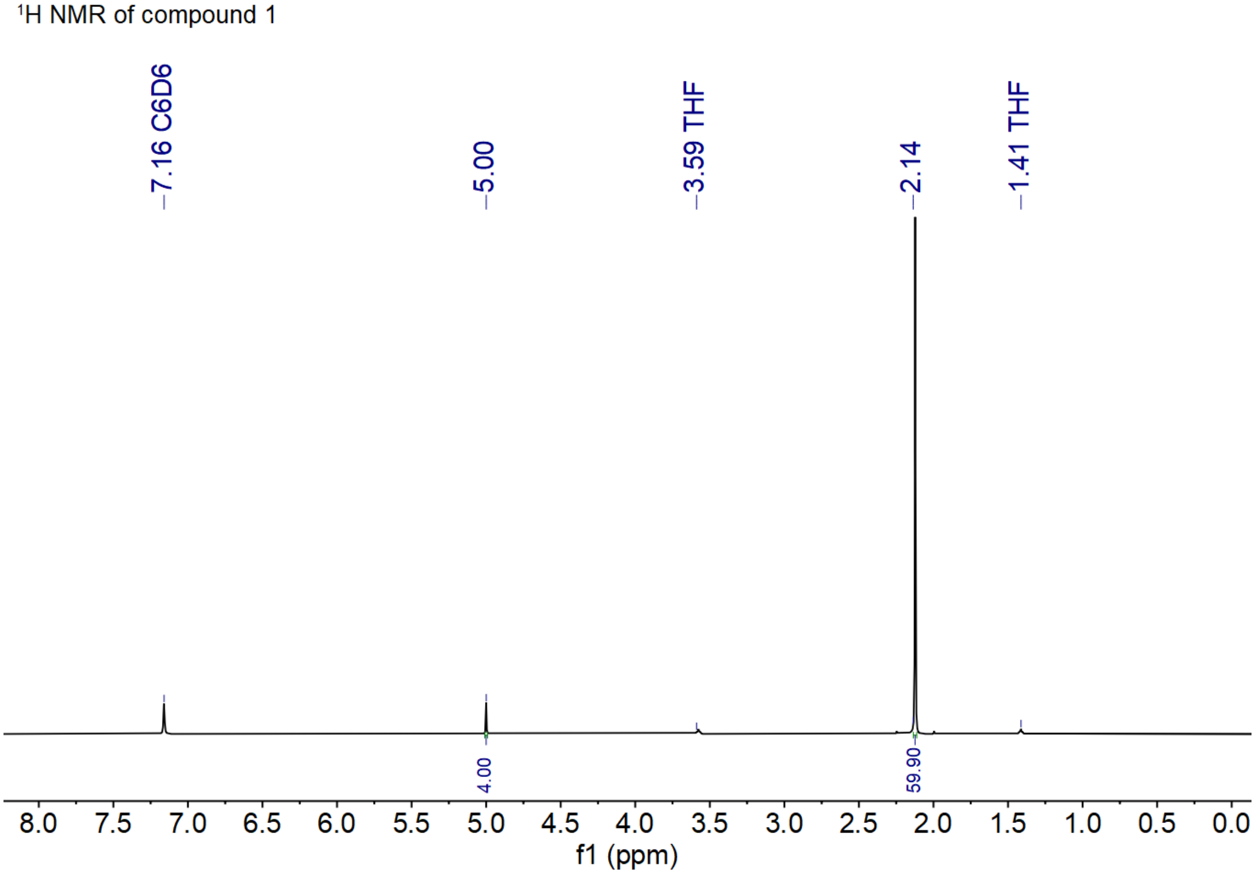

Figure S10. ^{1}H NMR spectrum of $(Cp^*{}_2Y)_2$(μ-tan), **1**, (500 MHz, ppm, C_6D_6, 25 °C): δ 5.00 (s, 4 H, $C_2(C\underline{H}N)_4$), 2.14 (s, 60 H, $C_5\underline{Me}_5$).

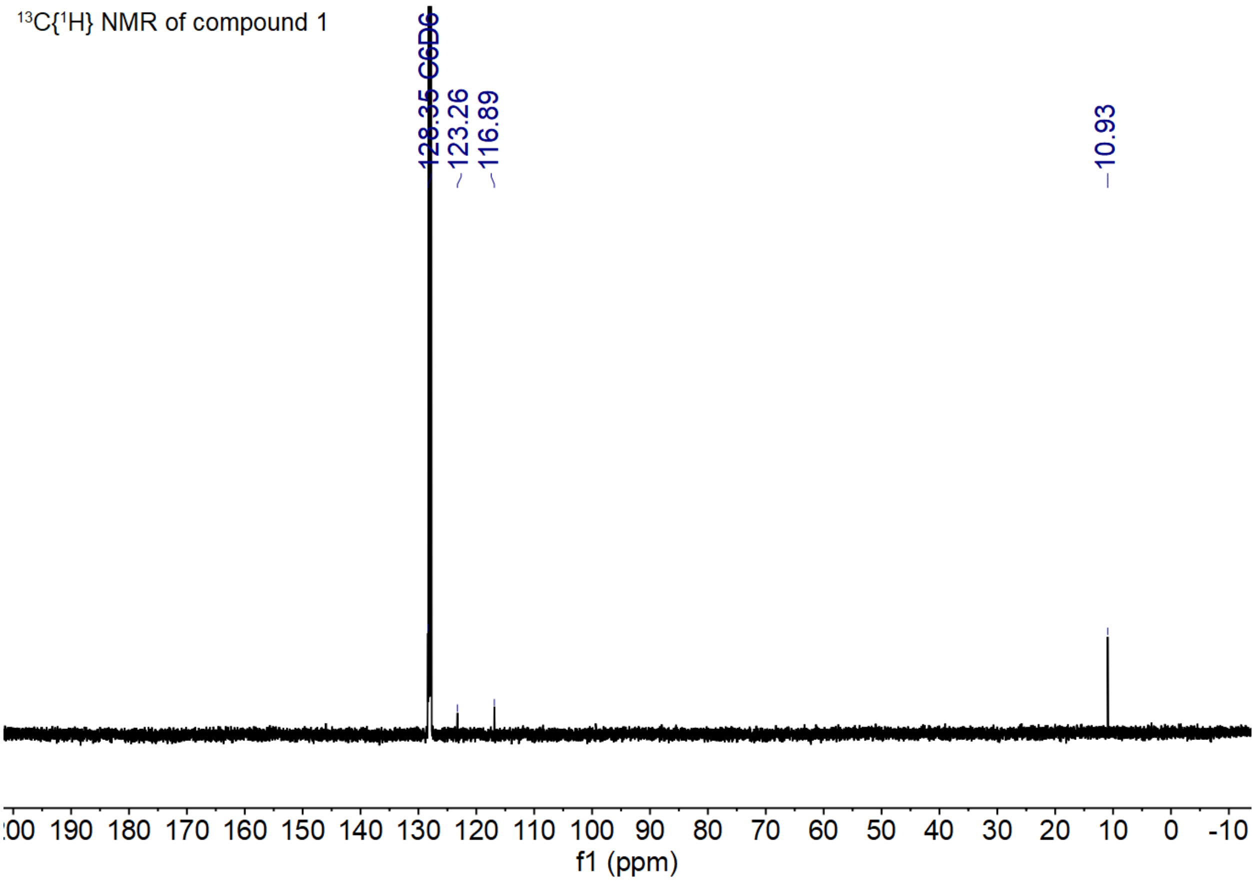

Figure S11. $^{13}C\{^{1}H\}$ NMR spectrum of $(Cp^*_2Y)_2(\mu\text{-tan})$, **1**, (126 MHz, ppm, C_6D_6, 25 °C) δ 123.3 ($C_2(\underline{C}HN)_4$), 116.9 ($\underline{C}_5Me_5$), 10.9 ($C_5\underline{Me}_5$).

3 IR Spectroscopy

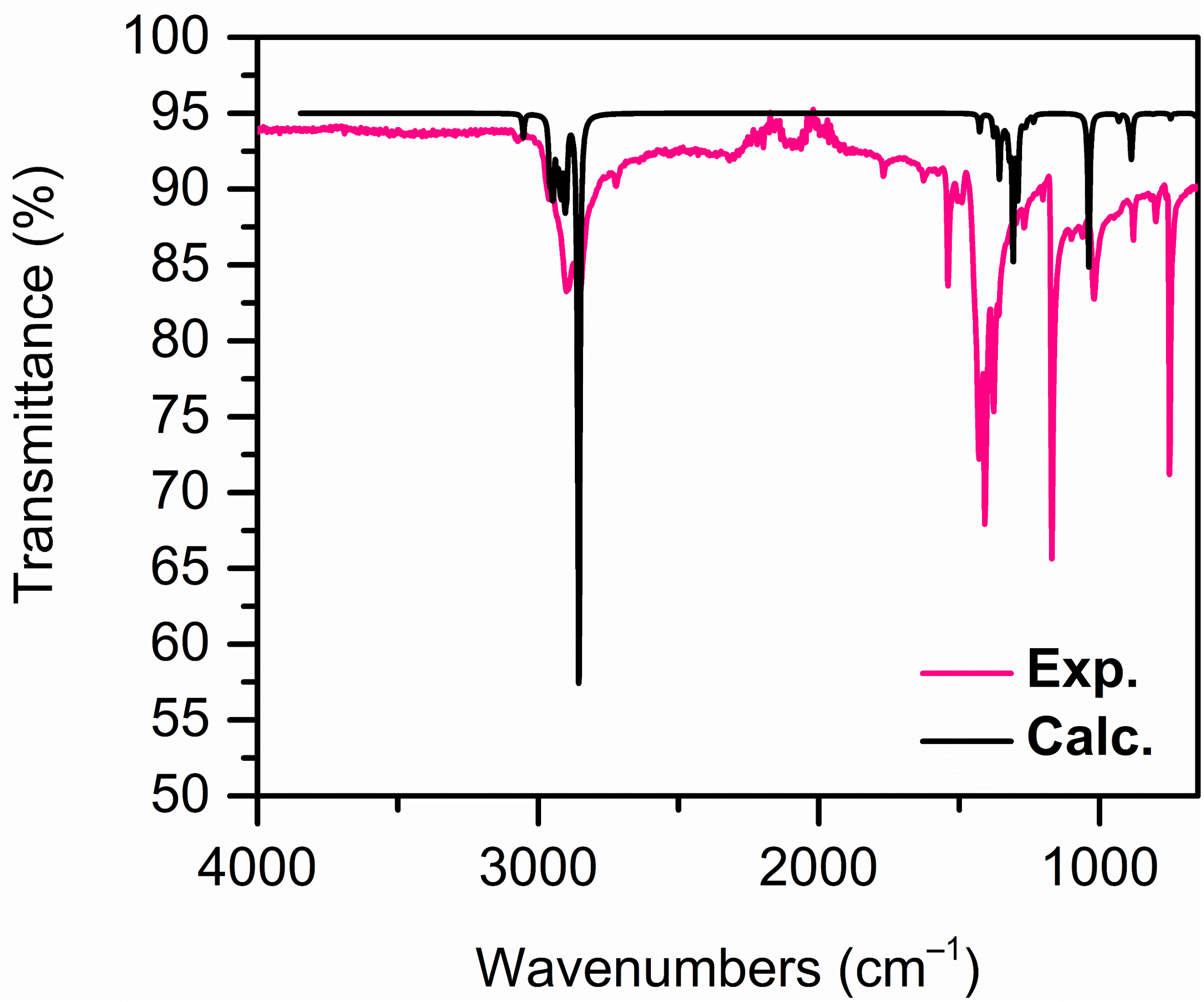

Figure S12. FTIR spectrum of $(Cp^*{}_2Y)_2$(μ-tan), **1**, (pink trace) collected on crushed crystalline solids under a nitrogen atmosphere. The DFT-calculated frequency spectrum for **1** is depicted in black. Calculated vibrational modes are shifted by –153 cm^{-1} to better match with the experimental spectrum.

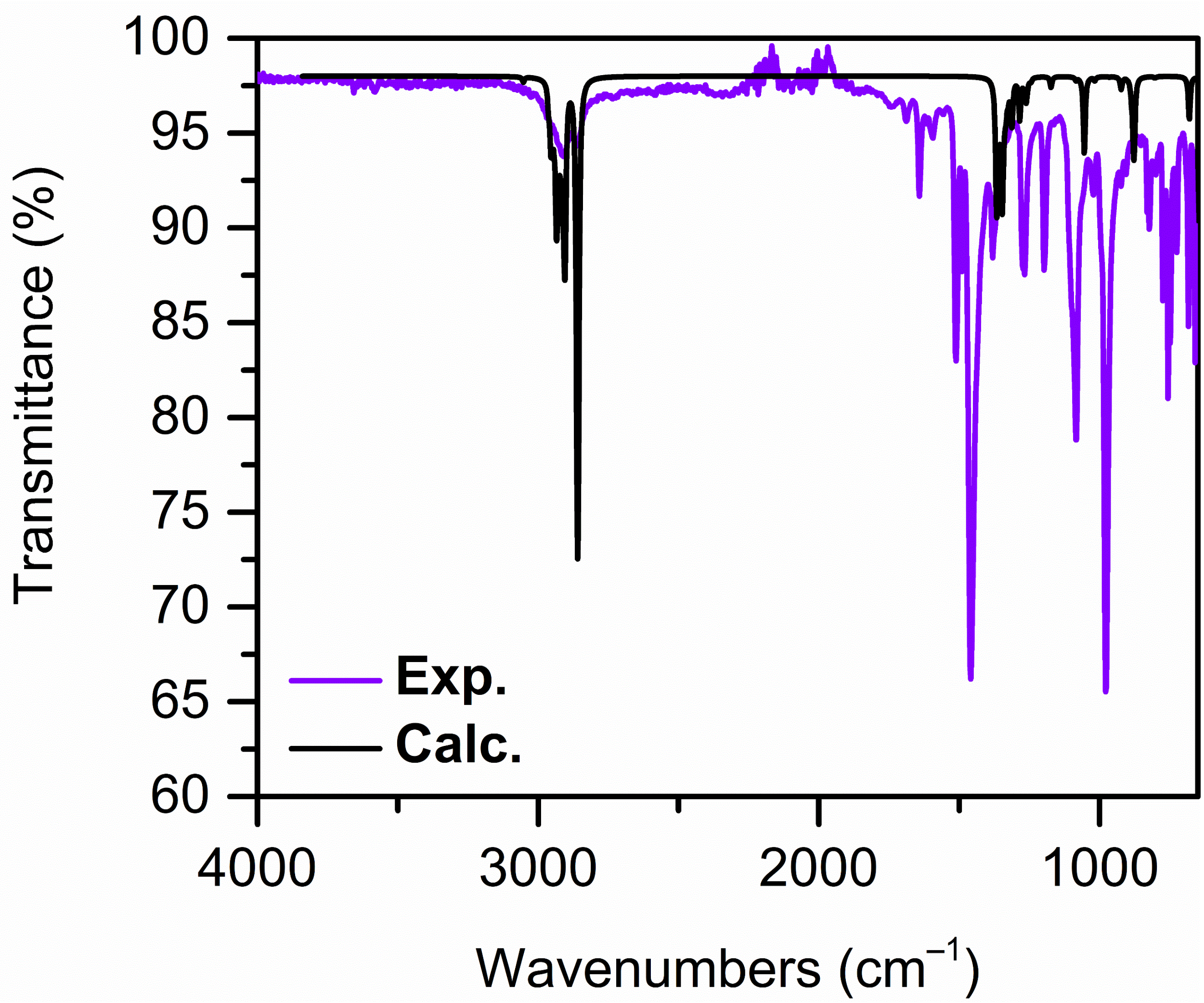

Figure S13. FTIR spectrum of [(Cp*$_2$Y)$_2$(μ-tan$^•$)][BArF$_{20}$], **2**, (violet trace) collected on crushed crystalline solids under a nitrogen atmosphere. The DFT-calculated frequency spectrum for **2** is depicted in black. Calculated vibrational modes are shifted by –160 cm^{-1} to better match with the experimental spectrum.

4 Cyclic Voltammetry

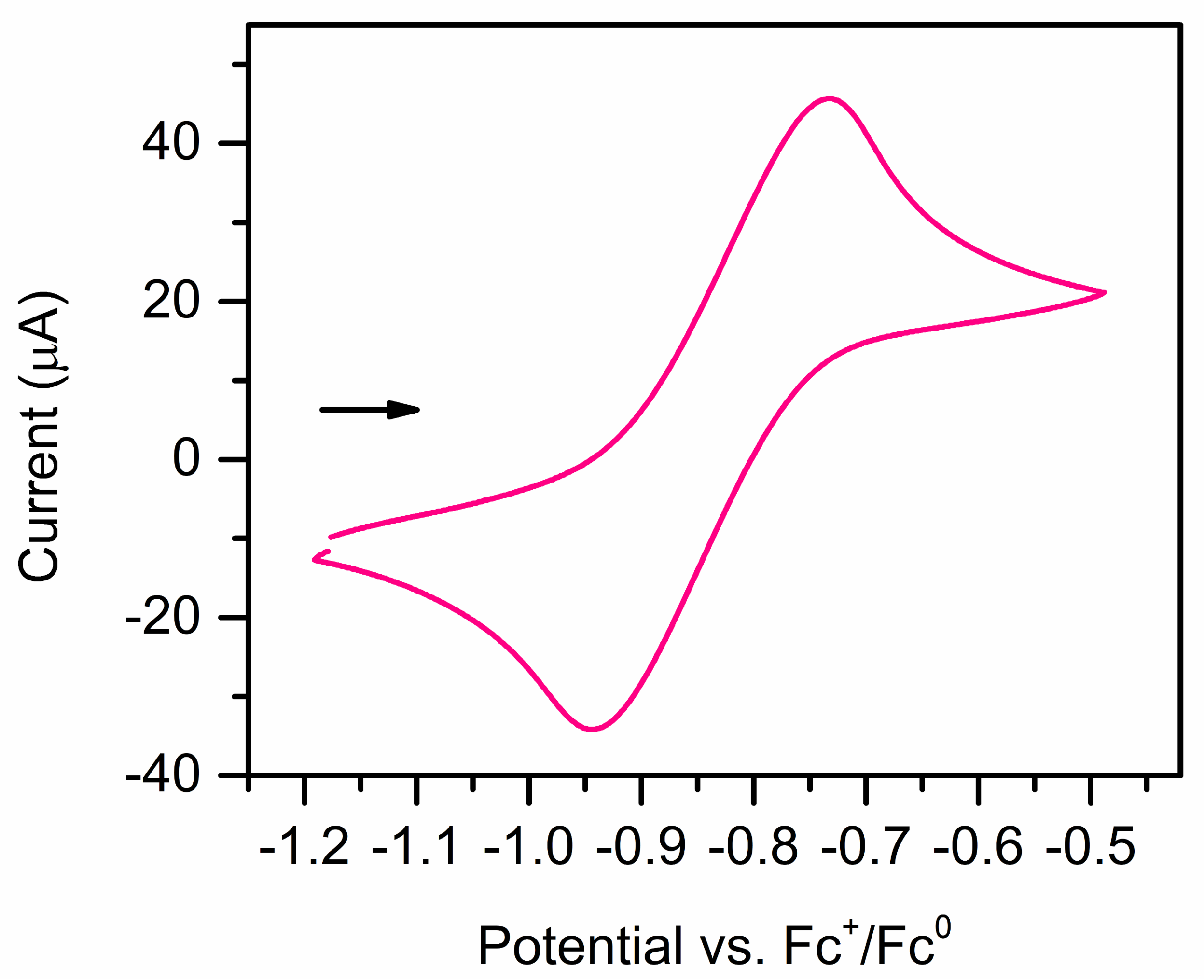

Figure S14. Quasi-reversible redox event observed at –0.84 V in the cyclic voltammogram of $(Cp^*_2Y)_2(\mu\text{-tan})$, **1**, vs. Fc. Measurements were conducted in 220 mM $[^nBu_4N][PF_6]$ electrolyte solution in 1,2-difluorobenzene with analyte concentration of 3 mM. Measurements were taken at 100 mV/s scan rates. (Fc. redox couple at 0.84 V.). Cyclic voltammogram was plotted following polarographic convention.

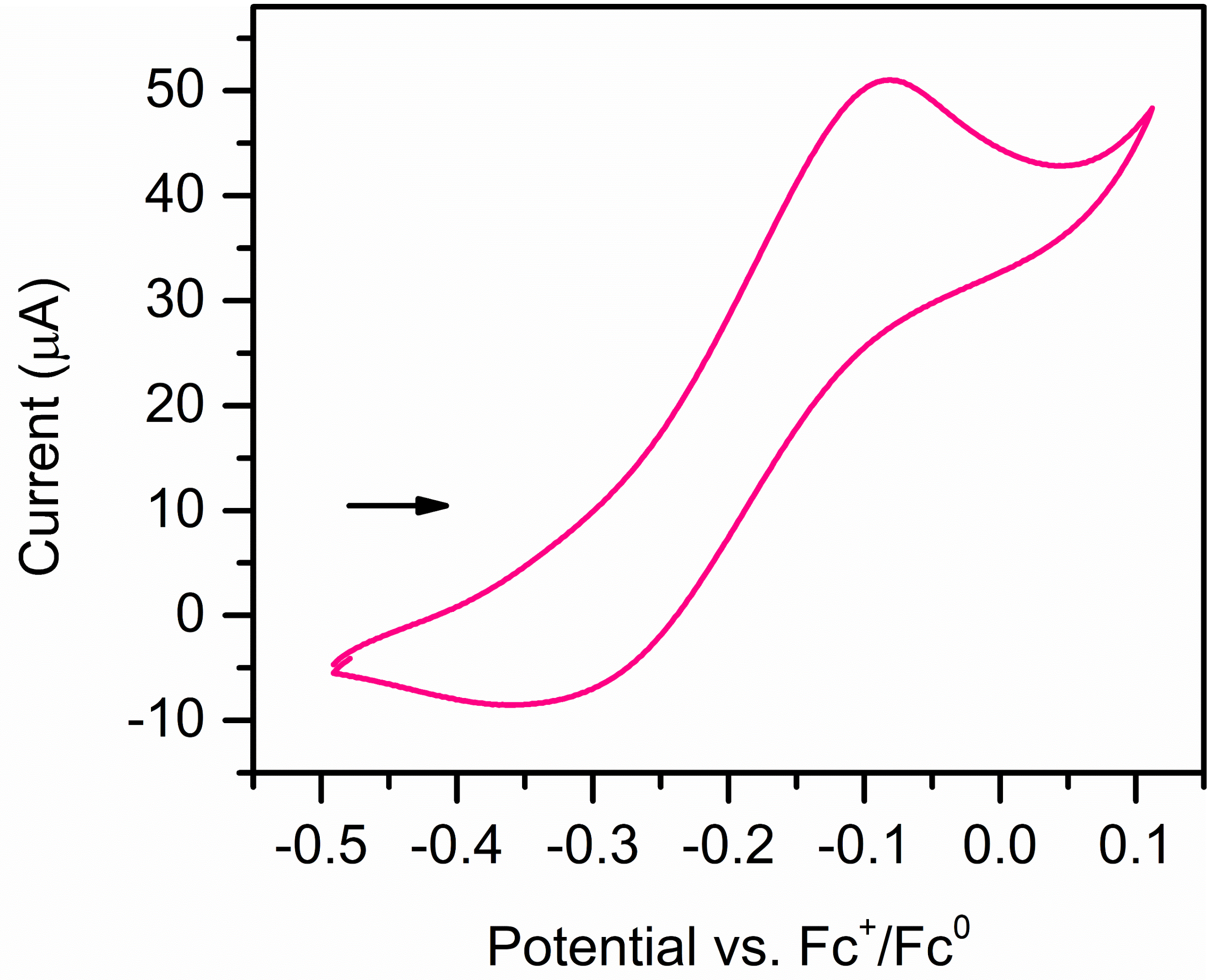

Figure S15. Quasi-reversible redox event observed at –0.18 V in the cyclic voltammogram of $(Cp^*_2Y)_2$(μ-tan), **1**, vs. Fc. Measurements were conducted in 220 mM $[^nBu_4N][PF_6]$ electrolyte solution in 1,2-difluorobenzene with analyte concentration of 3 mM. Measurements were taken at 100 mV/s scan rates. (Fc. redox couple at 0.84 V.). Cyclic voltammogram was plotted following polarographic convention.

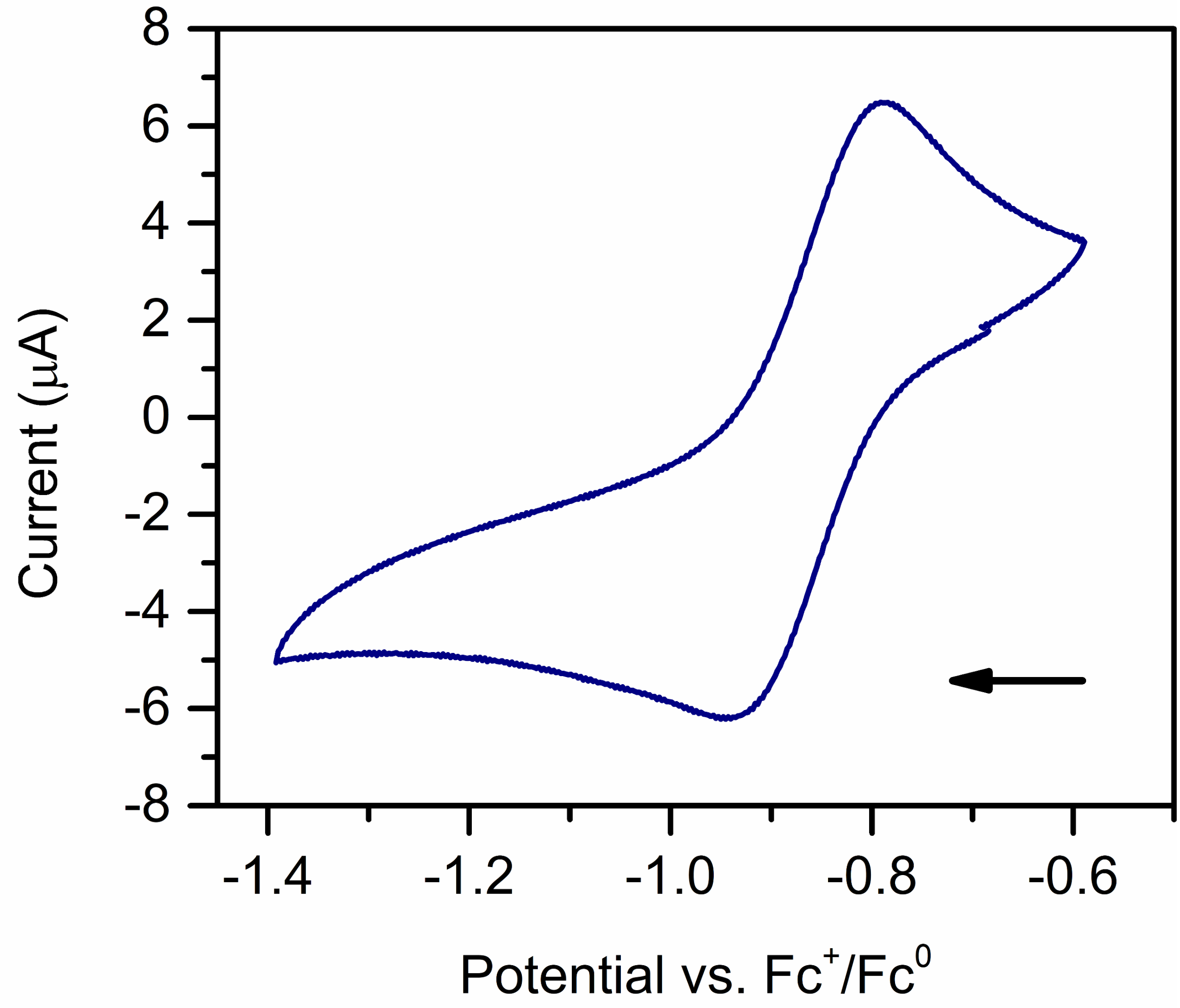

Figure S16. Quasi-reversible redox event observed at –0.89 V in the cyclic voltammogram of $[(Cp^*{}_2Y)_2(\mu\text{-tan}^\bullet)][BArF_{20}]$, **2**, vs. Fc. Measurements were conducted in 220 mM $[^nBu_4N][PF_6]$ electrolyte solution in 1,2-difluorobenzene with analyte concentration of 3 mM. Measurements were taken at 100 mV/s scan rates. (Fc. redox couple at 0.67 V.). Cyclic voltammogram was plotted following polarographic convention.

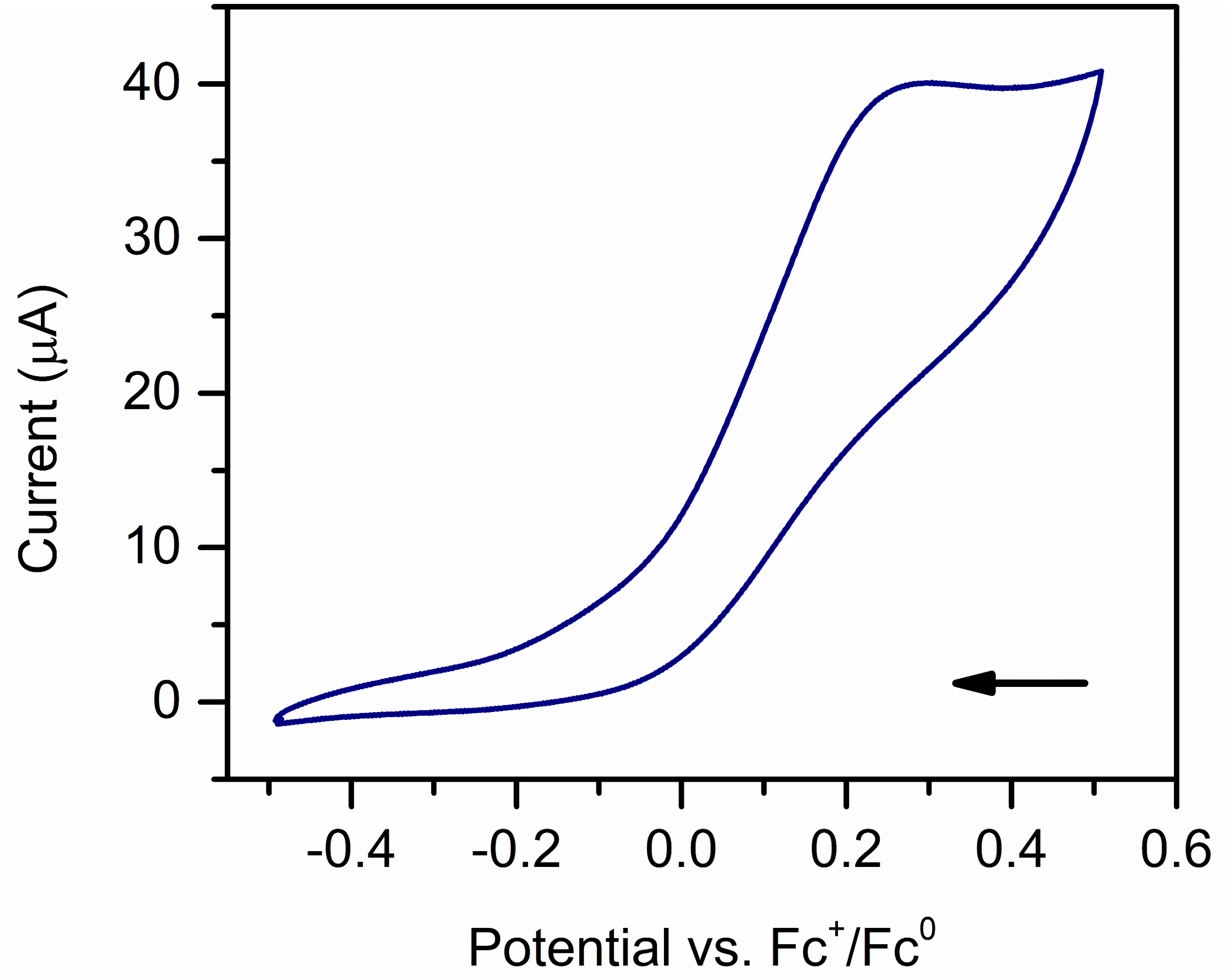

Figure S17. Quasi-reversible redox event observed at +0.16 V in the cyclic voltammogram of [(Cp*$_2$Y)$_2$(μ-tan$^•$)][BArF$_{20}$], **2**, vs. Fc. Measurements were conducted in 220 mM [^{n}Bu$_4$N][PF$_6$] electrolyte solution in 1,2-difluorobenzene with analyte concentration of 3 mM. Measurements were taken at 100 mV/s scan rates. (Fc. redox couple at 0.67 V.). Cyclic voltammogram was plotted following polarographic convention.

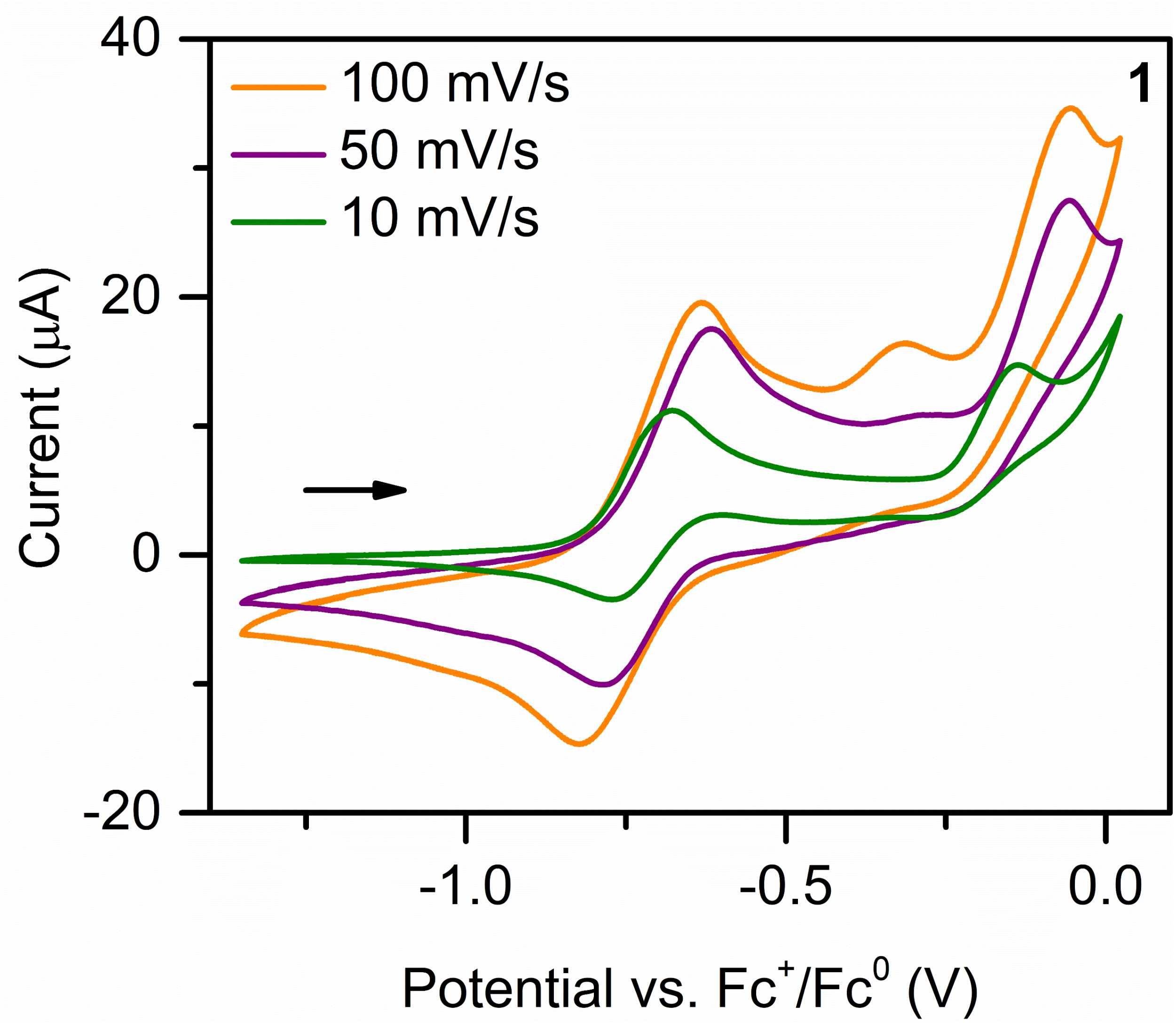

Figure S18. Cyclic voltammograms of $(Cp^*{}_2Y)_2$(μ-tan), **1** vs. Fc^+/Fc^0 redox couple, using variable scan rates. Cyclic voltammograms were collected in THF with 220 mM concentration of $[NBu_4][PF_6]$ supporting electrolyte and 3 mM analyte concentration. Arrow denotes the voltage scanning direction. (Fc. redox couple at 0.72 V). Cyclic voltammograms were plotted following polarographic convention.

5 DFT Calculations

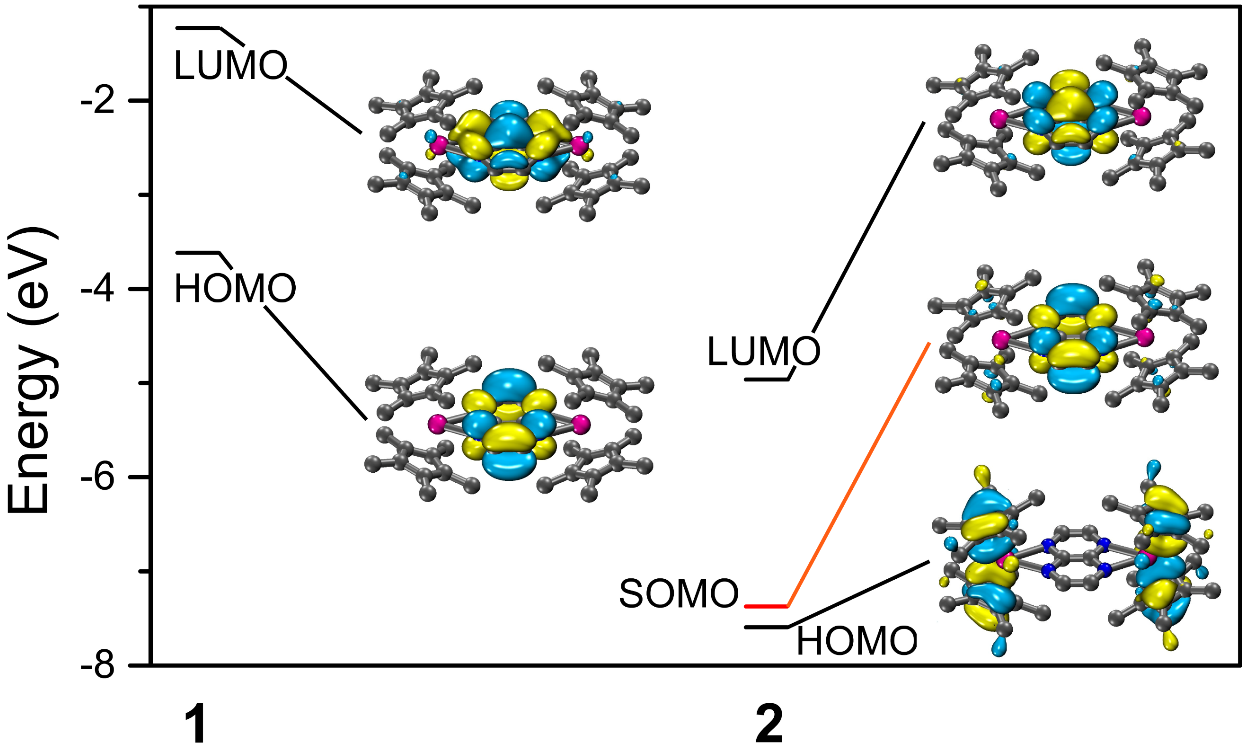

Figure S19. The DFT–calculated frontier molecular orbitals of $(Cp^*_2Y)_2$(μ-tan), **1**, (left) and $[(Cp^*_2Y)_2(\mu\text{-tan}^{\bullet})][BArF_{20}]$, **2**, (right). Pink, blue, and gray spheres represent Y, N, and C atoms. H atoms are omitted for clarity. Blue and yellow iso-surfaces represent different phases of molecular orbitals. DFT calculations were performed using uTPSSh functional at def2-TZVP level on all atoms with D3BJ dispersion correction. Isovalue for surfaces set at 0.03.

Table S2. Majority contributions of the TD-DFT–calculated transition states for $(Cp^*_2Y)_2(\mu\text{-tan})$, **1**, on the def2-TZVP level using the uB3LYP functional with D3BJ dispersion correction and THF implicit solvent model. The calculated excitation energies were empirically shifted by 0.37 eV. Isovalue for all depictions is 0.03. Oscillator strength cutoff used is 0.05 and contributions higher than 15% are shown. (HOMO = 194, LUMO = 195)

**λ (nm)**	**ν (cm^{-1})**	**Oscillator Strength**	**Occupied**	**Virtual**	**Weight (%)**
330.8	30226	0.32596	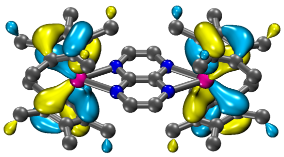 191α	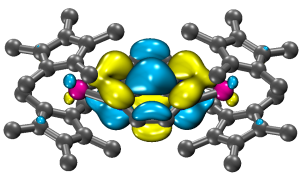 195α	50
			191β	195β	50
263.1	38009	0.25367	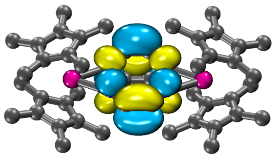 194α	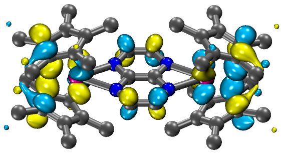 208α	42
			195β	208β	42
496.3	20148	0.22949	194α	195α	47
			194β	195β	47
260.3	38419	0.12940	194α	208α	42
			194β	208β	42
235.0	42548	0.11143	No individual contributions above 15%		
298.2	33538	0.10896	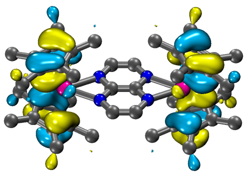 193α	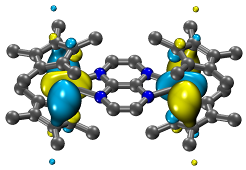 196α	26

			193β	196β	26
310.5	32207	0.08371	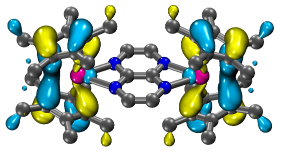 188α	195α	49
			188β	195β	49
252.3	39630	0.05466	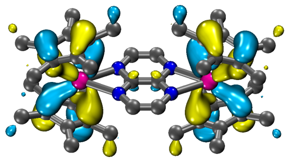 190α	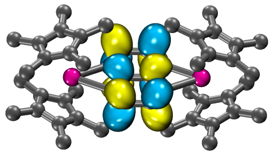 198α	49
			190β	198β	49

Table S3. Majority contributions of the TD-DFT–calculated transition states for [(Cp*$_2$Y)$_2$(μ-tan$^•$)][BArF$_{20}$], **2**, on the def2-TZVP level using the uB3LYP functional with D3BJ dispersion correction and DFB implicit solvent model. The calculated excitation energies were empirically shifted by 0.49 eV. Isovalue for all depictions is 0.03. Oscillator strength cutoff used is 0.05 and contributions higher than 15% are shown. (HOMO = 193, SOMO = 194, LUMO = 195)

λ (nm)	ν (cm^{-1})	Oscillator Strength	Occupied	Virtual	Weight (%)
320.4	31207	0.22103	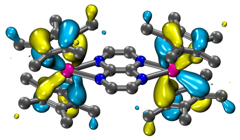 191β	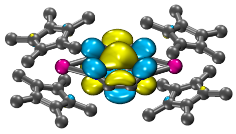 195β	95
305.3	32755	0.18914	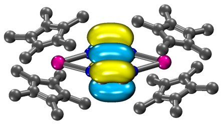 184β	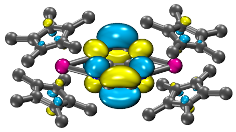 194β	48
363.2	27537	0.11127	191α	195α	97
300.0	33326	0.10294	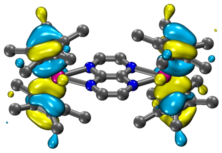 192α	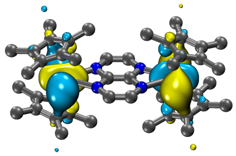 197α	41
			192β	197β	34
290.1	34473	0.10099	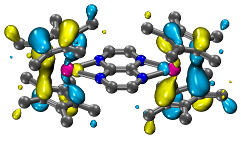 190β	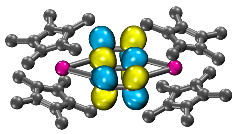 196β	49

224.7	44498	0.10093	190α	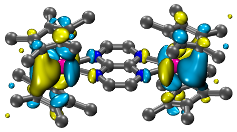 200α	32
			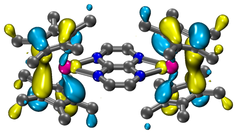 189β	200β	31
248.1	40313	0.09183	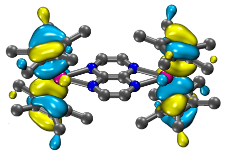 193α	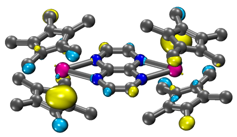 205α	34
			184β	196β	27
628.8	15904	0.07722	190β	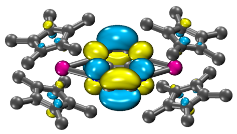 194β	99
216.2	46262	0.05457	No individual contributions above 15%		

Table S4. Atoms with Mulliken spin populations larger than 0.005 for optimized structure of $[(Cp^*_2Y)_2(\mu\text{-tan}^{\bullet})][BArF_{20}]$, **2**, and their respective spin population values. Calculation was conducted using uTPSSh functional at def2-TZVP level with D3BJ dispersion correction.

**Atoms**	**Spin Population**
N on tan ligand	0.167675, 0.164810, 0.167654, 0.167654
Peripheral C on tan ligand	0.112655, 0.111047, 0.111035, 0.112630
Bridgehead C on tan ligand	-0.038705, -0.038686
H on tan ligand	-0.007762, -0.008064, -0.008068, -0.007767
Y	-0.018063, -0.018059

Table S5. Cartesian coordinates of the geometry optimized structure of $(Cp^*_2Y)_2$(μ-tan), **1**, at def2-TZVP level using the uTPSSh functional and D3BJ dispersion correction. (Final single point energy = –2088.542605268362 Hartrees)

Y	6.332387	11.387579	13.073594
N	8.044269	12.991188	13.432360
N	8.215862	10.925521	14.442119
C	5.851915	11.095791	10.482633
C	7.916631	10.400569	11.222313
C	5.702075	9.796443	11.050096
C	7.219634	11.463896	10.589224
C	5.328285	12.560813	15.200614
C	5.020415	11.182554	15.353243
C	6.977220	9.374544	11.515996
C	4.582025	13.056586	14.096377
C	3.801258	11.988779	13.575834
C	4.079524	10.825187	14.351032
C	8.726893	12.154648	14.237198
C	8.574081	14.260095	13.258958
C	8.948743	10.073231	15.252807
C	9.954976	12.560101	14.857653
H	8.541482	9.082343	15.401811
C	10.107641	10.454747	15.836117
C	4.808652	11.863946	9.725139
C	9.393419	10.344563	11.455762
C	4.478337	8.933990	10.969319
C	7.833851	12.719314	10.045660
C	6.217340	13.357912	16.102234
H	10.667949	9.782109	16.471336
C	5.527469	10.280391	16.438563
C	7.295940	8.052028	12.150601
H	7.011483	12.735719	16.516898
H	5.654572	13.772596	16.946446
H	6.690337	14.190026	15.578431
C	4.576041	14.477141	13.611452
H	9.802835	11.343381	11.611554
H	9.913096	9.910582	10.593748
H	9.645637	9.744601	12.331423
H	3.802831	11.513107	9.955547
H	4.953873	11.753205	8.644300
H	4.844008	12.936036	9.940913
C	2.720376	12.124311	12.545755
H	5.592953	9.241823	16.102868
H	4.859120	10.294738	17.307479
H	6.519482	10.581020	16.779706
H	4.425347	8.220266	11.793363

H	4.478650	8.353338	10.039274
H	3.559495	9.522787	10.979715
H	8.137222	8.136003	12.842700
H	7.568253	7.302018	11.398994
H	6.443374	7.652066	12.706440
H	7.130888	13.556442	10.069905
H	8.140385	12.588414	9.001242
H	8.717308	13.012318	10.615441
H	5.534855	14.965292	13.800876
H	3.804881	15.069769	14.117103
H	4.374407	14.539974	12.538439
H	2.950275	12.897797	11.810881
H	1.771027	12.401145	13.019294
H	2.549730	11.191706	12.005239
C	3.368940	9.506155	14.265249
H	2.865814	9.381001	13.306517
H	2.605942	9.424446	15.048013
H	4.048898	8.658989	14.396383
C	9.732910	14.641657	13.842376
H	8.013478	14.932920	12.624198
Y	12.349782	13.326960	16.020740
N	10.637637	11.723540	15.662439
N	10.465983	13.789241	14.652758
H	10.140266	15.632484	13.693224
C	12.830846	13.618707	18.611585
C	10.765941	14.313912	17.872432
C	12.980525	14.918071	18.044107
C	11.463109	13.250582	18.505323
C	13.353499	12.153626	13.893633
C	13.661260	13.531889	13.740814
C	11.705259	15.339959	17.578534
C	14.100052	11.657991	14.997737
C	14.880882	12.725886	15.518004
C	14.602363	13.889397	14.742779
C	13.874302	12.850562	19.368821
C	9.289097	14.369913	17.639369
C	14.204265	15.780547	18.124595
C	10.849053	11.995132	19.048994
C	12.464253	11.356400	12.992300
C	13.153926	14.433927	12.655519
C	11.386362	16.662484	16.944038
C	14.106242	10.237472	15.482766
C	15.962039	12.590495	16.547812
C	15.312876	15.208484	14.828295
H	14.880058	13.201459	19.138219
H	13.729303	12.961243	20.449696

H	13.838950	11.778480	19.153002
H	8.879649	13.371093	17.483677
H	8.769641	14.803887	18.501520
H	9.036649	14.969876	16.763774
H	14.257050	16.494268	17.300532
H	14.204159	16.361208	19.054635
H	15.123116	15.191768	18.113989
H	11.552017	11.158011	19.024515
H	10.542803	12.125976	20.093503
H	9.965447	11.702148	18.479435
H	11.670024	11.978537	12.577716
H	13.026845	10.941600	12.148027
H	11.991361	10.524355	13.516309
H	13.088457	15.472521	12.991136
H	13.822094	14.419538	11.786464
H	12.161858	14.133217	12.314610
H	10.544884	16.578519	16.252175
H	11.114264	17.412484	17.695733
H	12.238772	17.062453	16.387963
H	13.147403	9.749253	15.293646
H	14.877289	9.644843	14.976942
H	14.308180	10.174728	16.555726
H	15.732398	11.817021	17.282778
H	16.911287	12.313720	16.074034
H	16.132751	13.523138	17.088239
H	15.816245	15.333731	15.786888
H	16.075662	15.290202	14.045325
H	14.632825	16.055596	14.697289

Table S6. Cartesian coordinates of the geometry optimized structure of [(Cp*$_2$Y)$_2$(μ-tan$^\bullet$)][BArF$_{20}$], **2**, at def2-TZVP level using the uTPSSh functional and D3BJ dispersion correction. (Final single point energy = -2088.362681876385 Hartrees)

Y	16.26319113123055	11.34896245293924	12.90321439882563
N	17.01082388013089	12.56283793797506	10.91698045707646
N	18.26147896616240	12.75684494269004	12.83784104503292
C	16.54467873043589	8.86466170944195	13.68645225811558
C	14.83067503008654	12.08984868298158	14.97005329717839
C	13.89378690985651	11.63915157557011	13.99432634206048
C	14.88929437017532	13.55722705489730	13.19578601207950
C	15.43849031866407	13.27715032666621	14.47781004383642
C	17.97666752489855	9.57565437374026	12.02764782441731
C	16.73396749855477	9.24048466622472	11.42243432983051
C	17.85591398643533	9.35177686783505	13.42827743568866
C	13.94277184085929	12.53806888809933	12.89224465163294
C	15.84886439149136	8.79892890935501	12.44280455392207
C	18.08810813331600	13.08359622903656	11.54039628772365
C	16.84990537958888	12.90198895623241	9.61832144878652
C	19.34751204258095	13.28311926399382	13.44736796139051
C	16.06233218573020	8.32425206693377	14.99946442050545
H	16.49843865709840	8.85619356042520	15.84694593968757
H	16.34169502008076	7.27023707714524	15.10269117732510
H	14.97685640642472	8.37939435270011	15.09048500065606
C	15.01370893179211	11.53032005474309	16.35058081174880
C	16.38754678007160	14.15150648923423	15.24191746878532
H	17.05841659365705	13.56840571096136	15.87845843978362
H	15.84051473248508	14.83476872616015	15.90029356175190
H	16.99989336021229	14.76335030657219	14.57678169845494
C	18.99601008594491	13.93004165523580	10.86467582589023
H	15.98958409535995	12.49264912319539	9.10583527840612
C	17.73651191578487	13.73069898110384	8.95778507402017
C	18.95018694384805	9.51278628030703	14.44281170728272
H	19.68769806895100	10.25270193283349	14.12333724818735
H	19.48655347711211	8.57055753756471	14.59717287033269
H	18.56308027231685	9.82030494584689	15.41797913610448
C	12.87459619135197	10.55776492841378	14.19085704692988
C	15.16087374916540	14.78526397730533	12.38204296317945
H	14.69270187716672	10.49067955247182	16.41164885959188
H	14.42244061421868	12.09723942301608	17.07762483539322
H	16.05479821540877	11.57945260516431	16.68184391319519
C	19.22583639913113	9.96924459046033	11.30148521504072
H	16.19535420320051	15.11786802330602	12.48567646483421
H	14.52400155553812	15.61501049147540	12.70642541139273
H	14.96558158657952	14.62318355485198	11.32048201165821
C	16.43529761055190	9.23991462650990	9.95239483994303

H	12.55597513738455	10.11643041168985	13.24595954637560
H	11.98106775936633	10.96865427873662	14.67313441784876
H	13.24328335553427	9.75544709115745	14.83125931479236
C	13.07765112118614	12.47798791564068	11.66642941316504
H	19.00067679852239	10.51823065665520	10.38403718849420
H	19.80484542910468	9.08626248199040	11.01150087330928
H	19.87643871040576	10.59235333522631	11.91788597760159
C	14.50413474902521	8.17829114890779	12.20416713290074
H	13.91805699528830	8.11689862483656	13.12005914636641
H	14.61939515371111	7.15866898042808	11.82133236553057
H	13.91638897568548	8.72976377883465	11.46518777631373
H	19.49883101138373	13.02997688777038	14.48804187769557
H	15.40084029744193	9.52771272935958	9.74419232233605
H	16.57741093923842	8.24151855676925	9.52514967923975
H	17.09245113709462	9.92018200640529	9.40698434925795
H	13.57299435484294	12.92206781746698	10.79899725843594
H	12.14244114460567	13.02811539321268	11.81521454699756
H	12.80611295056112	11.45142162216717	11.40914503044224
C	20.23423612698332	14.11164850813721	12.78677799534264
Y	20.82043618935120	15.66585575488948	9.50261550216039
N	20.07336565562280	14.45065332716539	11.48807692696410
N	18.82257178578070	14.25698473722559	9.56727964495224
H	17.58509497681407	13.98395027912798	7.91714879354341
H	21.09463064586436	14.52089273900787	13.29920844210581
C	20.54049949272455	18.15132145592640	8.72246074570389
C	22.25547115201317	14.92908790338499	7.43604962038493
C	23.19122815088409	15.37613313019797	8.41454727673245
C	22.19239637086729	13.45692019469678	9.20618997446980
C	21.64553308759719	13.74115343185995	7.92408833895180
C	19.10659818436719	17.43843914288806	10.37879393520603
C	20.34868884424421	17.77266039075935	10.98578408901187
C	19.22889893745510	17.66406366929259	8.97857695677588
C	23.13955160590629	14.47420227542750	9.51403672807105
C	21.23497489011502	18.21532421444056	9.96694227639142
C	21.02443045694360	18.69349369764367	7.41076572016402
C	22.07545037058367	15.49229340403332	6.05662499725500
C	24.21171183363241	16.45716922626927	8.22286562835404
C	21.91798545924745	12.22701928206866	10.01614112626291
C	20.69640760099965	12.87028950143907	7.15609726457329
C	17.85657703593406	17.04420895168369	11.10313467685025
C	20.64558976576261	17.77166154841666	12.45617480161289
C	18.13574045352241	17.50429371761024	7.96263163676755
C	24.00287077067585	14.52989175857975	10.74132492598613
C	22.57953085053999	18.83545455624424	10.20786425889141
H	20.58912099307922	18.16293471979735	6.56201733931309
H	20.74550196379364	19.74774574163913	7.30879829559662

H	22.10999389607895	18.63814767552077	7.32087349137824
H	22.39879577473810	16.53136034707012	5.99846046313486
H	22.66647579900789	14.92579147718636	5.32906088944354
H	21.03470708197288	15.44620097117290	5.72383751581496
H	24.52868257119002	16.89585110181740	9.16955995396003
H	25.10593219482393	16.04682483806469	7.74140941081747
H	23.84493930792926	17.26140050779113	7.58376156178993
H	20.88357070571579	11.89534220027230	9.90888319562836
H	22.55518824907621	11.39768955936381	9.69134720200092
H	22.11055263901368	12.38627952534294	11.07862943685107
H	20.02706773155701	13.45620644834823	6.52051406087814
H	21.24342453230052	12.18841983976855	6.49626639790369
H	20.08248579781399	12.25710175695317	7.81854005190859
H	18.08061887461682	16.49361972707271	12.01991076400927
H	17.27768647622297	17.92696295420373	11.39405278138620
H	17.20629059470917	16.42241488514765	10.48508047040450
H	21.67978775333118	17.48363531354617	12.66533491778042
H	20.50297240181516	18.76961990327307	12.88427554112217
H	19.98776406034967	17.09084529279009	13.00008421368890
H	17.39832736130469	16.76335208461234	8.27997751685757
H	17.59897090524455	18.44648421317617	7.80944463327186
H	18.52404361026551	17.19878001306812	6.98731729507504
H	23.50635227009668	14.08236553448986	11.60629322029943
H	24.93843614736288	13.98057145282118	10.59178823039612
H	24.27382499138968	15.55553143400123	11.00294742626900
H	23.16680562150376	18.89758511917384	9.29279202831968
H	22.46399988732743	19.85473432844834	10.59152830411421
H	23.16620342789690	18.28312710677419	10.94705732231491
